# Strengthening the pipeline of Aboriginal and Torres Strait Islander doctors into and through general practice training in Queensland, Australia: a scoping review

**DOI:** 10.3389/fmed.2026.1921854

**Published:** 2026-08-24

**Authors:** Dominic Orih, Robyn Grigg, James Flynn, Bradley Murphy

**Affiliations:** 1 The Royal Australian College of General Practitioners, Queensland Faculty, Brisbane, QLD, Australia; 2 College of Arts, Society and Education, The Cairns Institute, James Cook University, Cairns, QLD, Australia; 3 Mayne Academy Indigenous Health and Wellbeing, Faculty of Medicine, University of Queensland, Brisbane, QLD, Australia; 4 First Nations Medicine, Regional Medical Pathway, Central Queensland University, Rockhampton, QLD, Australia

**Keywords:** Aboriginal and Torres Strait Islander doctors, cultural safety, general practice training, general practitioners, health workforce equity, Indigenous health workforce, Queensland, scoping review

## Abstract

**Background:**

Aboriginal and Torres Strait Islander peoples experience significant health disparities relative to non-Indigenous Australians. Notwithstanding growing policy recognition of the link between Aboriginal and Torres Strait Islander health professionals and improved community health outcomes, their representation remains below parity in medical practice. This is particularly evident in general practice, where the general practitioner (GP) workforce shortfall intensifies nationally, and GP roles have become the hardest to fill across Aboriginal Community Controlled Health Organisations. This scoping review maps the evidence on strategies, programs, and frameworks for strengthening their participation, focusing on Queensland, where Aboriginal and Torres Strait Islander peoples form a comparatively large and geographically dispersed population.

**Methods:**

We followed the PRISMA Extension for Scoping Reviews (PRISMA-ScR) and the Joanna Briggs Institute methodology. Six databases (PubMed, CINAHL EBSCOhost, Scopus, Google Scholar, ProQuest, and Informit Indigenous Collection) and grey literature were searched, restricted to 2016–2026; one earlier eligible source from 2007 was retained via reference-list searching. After duplicate removal and staged screening, 27 sources were synthesised narratively across four domains: workforce data, programs and initiatives, international evidence, and barriers, enablers, and cultural safety frameworks.

**Results:**

Synthesis yielded five findings: (i) persistent underrepresentation despite sustained policy attention; (ii) cultural unsafety and racism as structural drivers of attrition; (iii) Indigenous self-determination in governance as a prerequisite for effectiveness; (iv) a financing gap indicating inadequate funding sustainability and disincentives at the hospital-to-general practice transition; and (v) a design gap between discrete interventions and the pipeline needed to address inequity at scale. Programs including the Indigenous General Practice Trainee Network, Joint Colleges Training Services, the Australian Indigenous Doctors' Association Specialist Trainee Support Program, the Royal Australian College of General Practitioners' Yagila Wadamba program, and the Remote Vocational Training Scheme, alongside Canadian and New Zealand models, offer transferable lessons.

**Conclusion:**

Strengthening the Aboriginal and Torres Strait Islander general practice pipeline in Queensland requires a longitudinal strategy combining culturally safe training, Aboriginal and Torres Strait Islander-led governance, sustainable multi-year funding, remuneration reform, and enhanced longitudinal data. Targeted action across every pipeline stage is needed to achieve workforce parity and improve outcomes in underserved communities.

## 1 Introduction

### 1.1 Background and health disparities

Aboriginal and Torres Strait Islander peoples in Australia experience greater health disparities than their non-Indigenous counterparts; and this contributes to lower life expectancy that has remained a constant concern for the Australian government (1–5). The Australian Institute of Health and Welfare (AIHW) (1) attributes approximately 35% of this health gap to social determinants such as employment, education, income, and housing; a further 30% is attributed to health risk factors like smoking; and roughly 35% are unexplained by these variables alone, which indicate factors that might be missing in the analysis, such as lack of access to affordable and culturally appropriate health care, connection to Country and language, and the effects of racism. These factors reflect the ongoing effects of colonisation, dispossession, and structural disadvantage that Aboriginal and Torres Strait Islander peoples continue to face (1, 5–10).

Martin and Reath (4) highlight that this life expectancy gap between Aboriginal and Torres Strait Islander peoples and non-Indigenous Australians has led the Australian Government to focus on strengthening the health workforce in terms of its size and quality by improving the recruitment, retention, training, and overall effectiveness of health practitioners working in Aboriginal and Torres Strait Islander health settings; and by actively increasing the number of health professionals who are Aboriginal or Torres Strait Islander peoples. According to Hayman et al. (11), ensuring that more Aboriginal and Torres Strait Islander doctors and nurses graduate and are able to work in their communities will increase the number of Aboriginal and Torres Strait Islander patients attending primary health care services, because they have greater trust in being cared for by their fellow Aboriginal and Torres Strait Islander peoples.

Karl Briscoe, CEO of the National Association of Aboriginal and Torres Strait Islander Health Workers and Practitioners, reinforced this view by asserting that improved health outcomes for Aboriginal and Torres Strait Islander people are demonstrably linked to the involvement of Aboriginal and Torres Strait Islander health professionals in the delivery of care within their communities [Briscoe, as cited in Liotta, (12)]. This is further corroborated by the National Association of Aboriginal and Torres Strait Islander Health Workers and Practitioners (NAATSIHWP) (13), whose guide for general practice notes that improved health and wellbeing outcomes of Aboriginal and Torres Strait Islander peoples across their life course are directly linked to Aboriginal and Torres Strait Islander health workforce practicing in their communities. As general practitioners (GPs) occupy a central role within the health workforce, the absence of Aboriginal and Torres Strait Islander GPs is tantamount to a workforce equity problem, which potentially has detrimental effects on Aboriginal and Torres Strait Islander health and safety (4). In Queensland, where Aboriginal and Torres Strait Islander peoples make up a larger share of the population and are concentrated in regional and remote communities, this workforce-equity problem is especially consequential.

### 1.2 The workforce gap: Queensland and national context

Aboriginal and Torres Strait Islander peoples comprise approximately 4.6% of Queensland's population and 3.8% of the total Australian population (14, 15). However, these population proportions stand in stark contrast to the far lower representation of Aboriginal and Torres Strait Islander peoples working across all stages of the medical training pipeline. Nationally, in 2022, only 191 Aboriginal and/or Torres Strait Islander doctors in training responded to the Medical Training Survey (16); by 2025, this number of survey respondents had dropped to 118 (17). While these figures represent survey respondents rather than the total number of Aboriginal and/or Torres Strait Islander doctors in training nationally, they nonetheless signal a deeply concerning level of representation across the medical training workforce. At the specialist level, the gap is even more significant. According to the Australian Indigenous Doctors' Association Specialist Trainee Support Program (AIDA STSP) Evaluation, only 0.3% of all medical specialists identify as Aboriginal and/or Torres Strait Islander (18). At the GP registrar level, according to the Workforce Insight Report of the Royal Australian College of General Practitioners (RACGP) (19), Aboriginal and Torres Strait Islander trainees make up only 1.5% of all Australian General Practice Training (AGPT) registrars nationally, and only 0.84% of RACGP members identify as Aboriginal and/or Torres Strait Islander (19). This is well below the population parity target of 3.8% (19). In Queensland, Aboriginal and Torres Strait Islander registrars numbered approximately nine out of a total GP registrar headcount of 1,289 in the first semester of 2025 (19). This accounts for 0.7% of the state's GP training cohort.

To paint a bigger picture of the extent to which the number of GPs does not meet projected workforce requirements, the RACGP (20) National Workforce Strategy 2025–2030 projects a current national GP shortfall of 3,010 full-time equivalent positions in 2025, growing to 6,400 by 2040 under baseline conditions. The document further reports that the intent to pursue general practice among final-year Australian medical students declined from 13 to 10.5% between 2021 and 2023 (20). A subsequent national analysis by the RACGP and Australian College of Rural and Remote Medicine (ACRRM) similarly recorded declining intent, yet found that actual entry into GP training runs at roughly double that level, with approximately one in three graduates progressing into the AGPT program (21). However, this gap between stated intent and actual entry cannot be assumed to hold evenly across all groups. For Aboriginal and Torres Strait Islander medical graduates, who already navigate additional barriers of racism, financial hardship, and limited peer support (18, 22, 23), this uneven conversion from intent to entry adds more structural headwinds that are bound to impact the health and wellbeing outcomes of Aboriginal and Torres Strait Islander communities as already established. The NAATSIHWP (13) noted that within general practice, the roles of Aboriginal and Torres Strait Islander Health Workers and Health Practitioners have remained largely unrealised; thus limiting the cultural safety and accessibility of primary care for Aboriginal and Torres Strait Islander patients. Achieving population parity of 4.6% at the Queensland level and 3.8% nationally would require different orders of change at each level. Reaching the national target (from 1.5 to 3.8% of AGPT registrars) would require an approximately 2.5-fold increase, whereas reaching parity in Queensland (from approximately 0.7%, i.e., nine of 1,289 registrars, to the Queensland population share of 4.6%) would require an approximately 6.5-fold increase in the number of Aboriginal and Torres Strait Islander GP registrars. This means that targeted promotion and pipeline investment are needed to strengthen the Aboriginal and Torres Strait Islander GP pipeline.

The geographical location of Aboriginal and Torres Strait Islander peoples in Queensland also highlights the urgency of ameliorating the issue of GP shortfall. The population of Aboriginal and Torres Strait Islanders in Queensland (roughly 237,000 people) is distributed across both urban and remote areas of the state (15). The highest proportional representation and most severe GP workforce shortages occur in remote communities (3, 11, 15). This paints a picture that reflects a broader national pattern in which the most significant health workforce challenge in general practice is distribution, whereby there is inadequate or non-existent service provision in rural and remote areas, particularly pronounced among Aboriginal and Torres Strait Islander communities that are of extreme disadvantage (24). Addressing the health needs of Queensland's most underserved communities will require strengthening the Aboriginal and Torres Strait Islander General Practice pipeline (3).

This is why this review, a Queensland Health–commissioned project, focuses on informing strategy for strengthening the Aboriginal and Torres Strait Islander general practice pipeline specifically within Queensland. The Queensland focus is warranted based on three considerations: (i) the commissioning and policy remit of Queensland Health; (ii) the distinctive geographic and demographic profile of Aboriginal and Torres Strait Islander communities in Queensland, including a substantial concentration of need in regional and remote communities; and (iii) the absence, to our knowledge, of any Queensland-specific synthesis on this topic. Consistent with this purpose, national Australian evidence and comparable international evidence from Canada and New Zealand are drawn upon throughout to offer transferable lessons to inform Queensland practice.

### 1.3 Policy context

In November 2024, the RACGP (25) released the Aboriginal and Torres Strait Islander Cultural and Health Training Framework. This document is structured around four interconnected elements: Cultural and Health Education and Training, Cultural Safety, Aboriginal and Torres Strait Islander Health Workforce Development, and the Aboriginal and Torres Strait Islander GP Training Pipeline. The Framework highlights that, as of June 2024, self-identified Aboriginal and Torres Strait Islander trainees constituted only 1.3% of all RACGP training enrolments and 0.39% of the total GP Fellows. While more recent data indicate a marginal increase to 1.5% of AGPT registrars by 2025 (19), this remains significantly below the national population parity target of 3.8% and the Fellowship gap of 0.39%. Thus, confirming that even marginal gains in registrar participation levels have not translated into parity, and that underrepresentation deepens markedly at the fellowship level. These figures must also be read against the broader national workforce target established by the National Aboriginal and Torres Strait Islander Health Workforce Strategic Framework and Implementation Plan 2021–2031 (26), which sets an overarching objective of Aboriginal and Torres Strait Islander people representing 3.43% of the total national health workforce by 2031. This target is premised on the evidence that reduced Aboriginal and Torres Strait Islander representation in health, contributes directly to reduced access to services for Aboriginal and Torres Strait Islander communities (26).

Complementing these national targets, the AGPT Program Performance and Outcomes Framework 2026–2030 (27) translates this broader policy ambition into program-specific accountability by setting a target for Aboriginal and Torres Strait Islander registrar enrolment, and mandating that registrars and supervisors complete cultural education. In addition to the AGPT Framework, the NAATSIHWP (13) released its Guide for General Practice in November 2025, developed in collaboration with and endorsed by the RACGP, to support the effective integration of Aboriginal and Torres Strait Islander Health Workers and Health Practitioners into general practice models of care. NAATSIHWP (13) views the document as a practical strategy for improving culturally safe service delivery for Aboriginal and Torres Strait Islander peoples. The RACGP (25) Cultural and Health Training Framework, NAATSIHWP (13) Guide for General Practice, National Workforce Strategic Framework (26), and AGPT Performance and Outcomes Framework (27), reflect a growing and multi-layered policy recognition that closing the gap in health outcomes requires not only increasing the number of Aboriginal and Torres Strait Islander GPs in training, but also embedding Aboriginal and Torres Strait Islander health professionals more broadly across the primary care workforce. This needs to be done within accountable, measurable, and adequately resourced national systems. Our scoping review treats these documents collectively as both evidence and benchmarks. The persistence of this workforce gap, set against the targets and benchmarks established by these documents and the broader policy context outlined above, gives rise to the research questions that underlie this scoping review. Although these policy instruments are national in scope, their implementation depends on state-level systems. In Queensland, this makes the translation of national targets into local training, governance, and community structures the central concern of the present review.

### 1.4 Research questions

#### 1.4.1 Primary research question

What strategies, programs, and frameworks have been implemented or proposed to strengthen the pipeline of Aboriginal and Torres Strait Islander doctors into and through general practice training in Queensland, drawing on national Australian and international evidence?

#### 1.4.2 Secondary questions

1. What is the current state of evidence on Aboriginal and Torres Strait Islander representation in general practice training and the broader medical workforce relevant to Queensland, including national Australian data?
2. What programs and interventions have been implemented or evaluated to support Aboriginal and Torres Strait Islander doctors' entry into and progression through general practice training?
3. What barriers and enablers affect Aboriginal and Torres Strait Islander doctors' pathways into general practice training across the post-graduation pipeline?
4. What lessons can be drawn from Australian and international evidence to inform strategies for strengthening the pipelines in Queensland?

## 2 Methods

### 2.1 Protocol, design, and registration

The aim of this review is to describe and synthesise existing evidence on enhancing the pipeline of Aboriginal and Torres Strait Islander doctors into general practice. Scoping reviews are suited for mapping heterogeneous evidence, identifying gaps, and informing policy directions (28, 29), which align well with the current state of evidence on Aboriginal and Torres Strait Islander GP workforce development. Based on this, we opted for this review approach instead of a systematic literature review. We followed the current guidelines for the Preferred Reporting Items for Systematic Reviews and Meta-Analysis Extension for Scoping Reviews (PRISMA-ScR) (29), informed by the broader PRISMA 2020 reporting framework (30), and guided by the Joanna Briggs Institute (JBI) methodology (28). The protocol was registered with the Open Science Framework (OSF).

### 2.2 Eligibility criteria

We defined the eligibility criteria using the Population-Concept-Context (PCC) framework, as itemised in Table 1. The population included Aboriginal and Torres Strait Islander doctors, medical trainees, and GP registrars in Australia, as well as Indigenous physicians in comparable international jurisdictions (Canada and New Zealand). The concept includes barriers, enablers, programs, interventions, and policies affecting pathways into and through general practice training. It also includes workforce representation, cultural safety frameworks, racism and discrimination, and reforms in admissions and selection pathways. The context focuses on post-graduation medical training settings in Australia, Canada, and New Zealand. The eligibility criteria deliberately admit national Australian and comparable international evidence (from Canada and New Zealand) for the purpose of informing the Queensland context, rather than restricting inclusion to Queensland-generated studies alone, which are few.

**Table 1:** Population-concept-context (PCC) framework.

Component	Definition	Key elements
Population	Aboriginal and Torres Strait Islander doctors, medical trainees, and GP registrars in Australia; Indigenous physicians and medical learners in comparable international jurisdictions	•Aboriginal and Torres Strait Islander intern doctors and prevocational trainees •GP registrars and recently fellowed GPs (within 5 years) •Non-GP specialist trainees identifying as Aboriginal and/or Torres Strait Islander •Key stakeholders (training organisations, supervisors, Aboriginal Community Controlled Health Organisations, policy bodies) •Indigenous physicians and trainees in Canada and New Zealand
Concept	Barriers, enablers, programs, interventions, and policies affecting pathways into and through general practice training and specialist training	•Workforce representation, trends and equity •Programs and initiatives supporting GP training participation •Barriers and enablers to progression and retention •Cultural safety frameworks and implementation •Racism, discrimination and mentorship •Admissions and selection pathway reforms
Context	Post-graduation medical training settings in Australia, Canada, and New Zealand	•Australian GP training programs (AGPT, RVTS, and ACRRM) •Queensland health and training systems •Aboriginal Community Controlled Health Organisations (ACCHOs) •Medical colleges and training bodies •Indigenous-led programs and organisations

GP, general practitioner; AGPT, Australian General Practice Training; ACRRM, Australian College of Rural and Remote Medicine; RVTS, Remote Vocational Training Scheme; ACCHOs, Aboriginal Community Controlled Health Organisations.

We agreed on our inclusion criteria to involve literature addressing Aboriginal and Torres Strait Islander (or internationally, First Nations, Māori) doctors, medical graduates, interns, residents, registrars, or recently fellowed GPs (within 5 years of fellowship). We focused on general practice, primary care, or rural generalist training, and contextualised the search in Australian, Canadian, or New Zealand settings. To delineate the type of sources included, peer-reviewed articles, government reports, program evaluations, and grey literature were included in our literature search. We restricted the database and grey-literature searches to publications from January 2016 to March 2026, with one earlier eligible source (2007) retained through reference-list searching (see Section 2.3). The rationale for this was to capture a sufficient body of evidence for synthesis, given the relative paucity of literature on Aboriginal and Torres Strait Islander GP workforce development, particularly in Queensland; and also to ensure that we capture contemporary GP training reforms. The exclusion criteria included literature that focused exclusively on non-Indigenous populations, non-medical health workforces, conference abstracts without full-text data, and non-English publications without translation. English-language restriction was applied due to resource constraints for language translation and because our primary focus was on Australia, Canada, and New Zealand. Table 2 presents the eligibility criteria.

**Table 2:** Eligibility criteria for inclusion and exclusion.

Criterion	Inclusion	Exclusion
Population	Aboriginal and Torres Strait Islander doctors, medical graduates, interns, residents, registrars, or recently fellowed GPs (within 5 years of fellowship); First Nations or Māori physicians and medical trainees in comparable international jurisdictions	Studies focused exclusively on non-Indigenous populations
Concept	General practice, primary care, or rural generalist training pathways, programs, barriers, enablers, and policies	Studies focused exclusively on non-medical health workforces (e.g., nursing, allied health only)
Context	Australian, Canadian, or New Zealand settings	Studies set outside Australia, Canada, or New Zealand without relevance to comparable Indigenous medical workforce contexts
Study types	Peer-reviewed articles, government reports, program evaluations, and grey literature	Conference abstracts without full-text data
Language	English-language publications only	Non-English publications
Publication period	January 2016 to March 2026 (database and grey-literature searches); earlier 2007 source admitted only via reference-list (citation) searching	Publications before 2016 (other than the 2007 source identified through reference-list searching)

### 2.3 Search strategy

We conducted our searches between March and April 2026 across six databases (PubMed, CINAHL EBSCOhost, Scopus, Google Scholar, ProQuest, INFORMIT Indigenous Collection), and Gray Literature, yielding a total of 1,170 records. The searches combined the terms of population, concept, and context with Boolean operators (“OR” and “AND”). We adapted the search strings to meet the syntax and indexing requirements of each database searched. In every string, Indigeneity terms were combined with general practice and training terms. Jurisdictional delimitation to Australia, Canada, and New Zealand was achieved principally through the Indigeneity terms themselves (for example, “Aboriginal Australian,” “Torres Strait Islander,” “Maori”) and was applied in full at the eligibility-screening stage, rather than as a separate Boolean facet within every string. General practice was not searched as a standalone concept, as this would have returned a large volume of literature unrelated to Indigenous workforce, cultural safety, or pipeline programs. The six bibliographic database searches were applied to peer-reviewed literature and, in aggregate, returned 1,104 records. The complete database-specific search strings, filters (date, language, document type, and subject-area or indexing limits), and record counts are provided in Supplementary Table S1. Gray literature was searched separately and returned 66 records, giving 1,170 records in total before de-duplication. Gray-literature sources were identified through targeted searching of the websites and publication repositories of relevant organisations, including the RACGP, ACRRM, Australian Indigenous Doctors' Association (AIDA), Indigenous General Practice Trainee Network (IGPTN), Joint Colleges Training Services (JCTS), National Aboriginal Community Controlled Health Organisation (NACCHO), the Medical Board of Australia and AHPRA (the Medical Training Survey), the Australian Department of Health, Disability and Ageing, and equivalent Canadian and New Zealand bodies. These organisational searches were supplemented by targeted Google and Google Scholar searching and by reference-list (citation) searching of included sources.

All searches, across both the bibliographic databases and grey literature, were restricted to the period January 2016 to March 2026. One additional eligible source published in 2007 (Anderson and Lavallee) was identified through reference-list searching and retained, given the paucity of literature in the Queensland and Australian Aboriginal and Torres Strait Islander general-practice-training context, which accounts for the 2007–2026 span of the included sources. Table 3 presents the search string executed in Scopus, together with the filters applied and the number of records retrieved, as an illustration of the strategy applied across the databases searched. The complete database-specific strings, filters, and record counts are provided in Supplementary Table S1.

**Table 3:** Executed search string on scopus.

Element	Detail
Database	Scopus (Elsevier)
Field	ALL
Search string	((“Aboriginal Australian” OR “Torres Strait Islander” OR “Indigenous Australian doctor” OR “First Nations physician” OR “Indigenous physician” OR “Maori doctor” OR “Indigenous medical trainee” OR “Aboriginal doctor” OR “Indigenous doctor” OR “Aboriginal medical graduate”) AND (“general practice training” OR “GP training” OR “general practitioner training” OR “vocational training” OR “primary care training”) AND (“workforce” OR “cultural safety” OR “racism” OR “barriers” OR “enablers” OR “retention” OR “recruitment” OR “mentorship” OR “leadership” OR “pipeline”))
Filters	Date 2016–2026; English; Document type Article, Review; Subject areas Medicine, Health Professions, Multidisciplinary, Social Sciences
Date executed	11 March 2026
Records retrieved	79

Presented as representative of the strategy. Complete strings, filters, and counts for all databases are provided in Supplementary Table S1.

### 2.4 Selection of papers and data management

**Figure 1:**
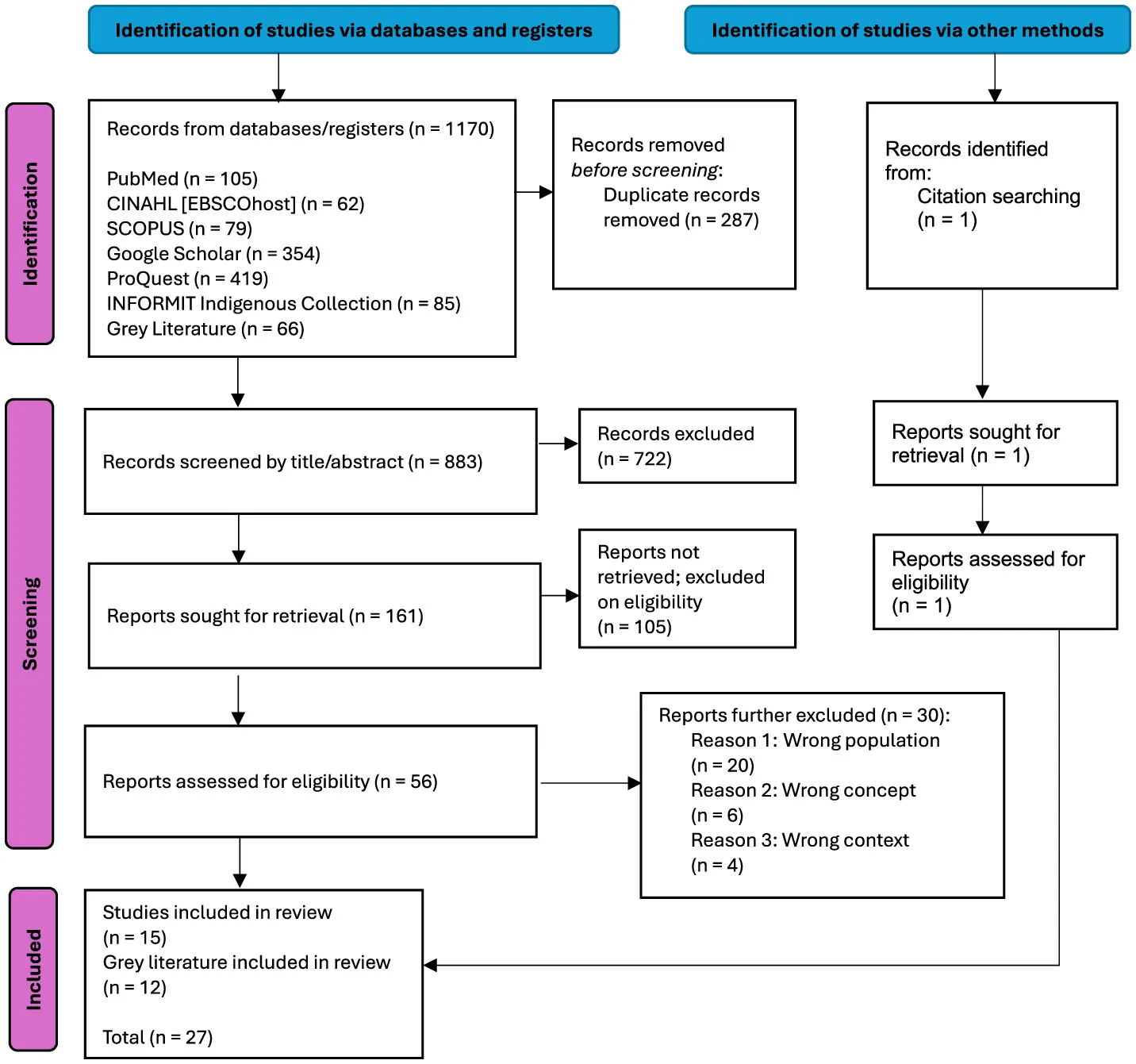
PRISMA flow diagram. *Source:* Page et al. (30). For more information, visit www.prisma-statement.org/.

The first author (DO) independently screened all peer-reviewed articles, while the second author (RG) independently screened the grey literature and organisational records. All 1,170 records identified from the databases and grey literature were exported to EndNote X20 by the first author (DO). After removing 287 duplicates, 883 records were screened for eligibility. To ensure consistency, DO cross-checked a randomly selected 20% of the grey literature screened by RG, and RG cross-checked a randomly selected 20% of the peer-reviewed articles screened by DO. Any disagreements were adjudicated by the third and fourth authors (JF and BM). After screening the titles and abstracts of the selected materials, the full texts of 161 records were retrieved for an in-depth review and to ascertain their eligibility for inclusion. Of these 161 records, 105 were further excluded because they did not meet the inclusion criteria upon full-text review. The Full-text assessment of the remaining 56 reports resulted in 30 further exclusions. The reasons for exclusion included wrong population (*n* = 20), wrong concept (*n* = 6), and wrong context (*n* = 4). After this exclusion process, 26 records from database and grey literature searches, together with one additional source identified through reference-list (citation) searching, were included in this scoping review (27 in total). The selection process is illustrated in Figure 1 using the PRISMA flow diagram (30).

### 2.5 Data charting and synthesis

A charting form was used by the authors (DO and RG) to capture and extract relevant data from all selected materials in a tabular form (see Supplementary material). The extracted variables included author(s), year of publication, and country; population; topic domain; intervention/program; outcomes; key findings; limitations; and relevance to Queensland. Peer-reviewed literature data were extracted by DO and verified by RG independently, and grey literature and organisational record data were extracted by RG and verified by DO independently. Discrepancies were resolved among the authors through iterative discussions to reach a consensus. The included papers were examined according to their relevance to our research questions. In line with the JBI scoping review methodology, a formal quality appraisal of individual studies or documents was not conducted. This is because scoping reviews aim to map the breadth of available evidence rather than produce a synthesised and clinically definitive answer to a specific question (28, 29). As a result, evaluating the methodological limitations or risk of bias of the included sources is generally not undertaken unless the specific aims of the review require it (28). Accordingly, we used narrative synthesis to present our findings across four topic domains, given the heterogeneity of the study designs. The “relevance to Queensland” field of the charting form in the supplementary material was used to draw out, for each source, the applicability of national and international evidence to the Queensland context, ensuring that the synthesis remained anchored to the review's Queensland focus.

## 3 Results

### 3.1 Characteristics of included sources

The 27 records included in the review comprised 15 peer-reviewed journal articles and 12 grey literature sources. As already delineated, the literature included government reports, program evaluations, organisational annual reports, policy frameworks, and policy submissions, published between 2007 and 2026. The included sources originated primarily from Australia (*n* = 18), with comparators from Canada (*n* = 5) and New Zealand (*n* = 3). One narrative review addressed multiple international jurisdictions; therefore we classified it separately as “International” (*n* = 1). Study designs across the full sample were diverse, ranging from mixed-methods evaluations and cross-sectional surveys to qualitative studies, narrative reviews, program descriptions, self-assessment reports, organisational annual reports, policy analyses, and policy submissions. The topics discussed were thematically arranged according to the following domains: Topic domain 1 = Workforce Data; Topic domain 2 = Programs and Initiatives; Topic domain 3 = International Evidence; and Topic domain 4 = Barriers, Enablers, and Cultural Safety Frameworks. Table 4 presents the characteristics of the 27 included sources in this review.

**Table 4:** Characteristics of included papers (*n* = 27).

#	References	Title (abbreviated)	Country	Design	Topic domain(s)
1	Anderson and Lavallee (44)	Development of the First Nations, Inuit and Métis medical workforce	Canada	Policy/program description	2, 3
2	Australian Indigenous Doctors' Association (AIDA) (31)	Growing the number of Aboriginal and Torres Strait Islander medical specialists: self-assessments by specialist medical colleges	Australia	Self-assessment report/policy document	1, 2, 3, 4
3	Black et al. (41)	Indigenous medical students on a postgraduate Indigenous admissions pathway	Canada	Qualitative study	2, 3
4	Burm et al. (48)	Navigating the burden of proof and responsibility	Canada	Narrative inquiry	3, 4
5	Cormack et al. (47)	Māori medical student and physician exposure to racism	New Zealand	Cross-sectional survey	3, 4
6	Curtis et al. (46)	Refining definitions of cultural safety and cultural competency	New Zealand	Conceptual/analytical	3, 4
7	Department of Health, Disability and Ageing (27)	AGPT Program Performance and Outcomes Framework 2026–2030	Australia	Policy/program framework	2, 4
8	Giddings et al. (34)	Australia's Remote Vocational Training Scheme	Australia	Perspective/program overview	2
9	Giddings et al. (35)	Retention of doctors in remote, rural and First Nations communities	Australia	Perspective/program evaluation synthesis	2
10	Indigenous General Practice Trainee Network (IGPTN) (22)	IGPTN Annual Report 2024–2025	Australia	Organisational annual report/financial report	1, 2, 4
11	Joint Colleges Training Services (JCTS) (39)	JCTS Strategic Plan 2025	Australia	Organisational strategic plan	2, 4
12	Koea et al. (42)	Affirmative action in postgraduate medical and surgical training	International	Narrative review	2, 3
13	Martin and Reath (4)	General practice training in Aboriginal and Torres Strait Islander health: GPET Framework	Australia	Program description/evaluation	2, 3
14	Medical Board Australia and AHPRA (17)	Medical Training Survey 2025: Aboriginal and Torres Strait Islander doctors in training	Australia	Cross-sectional survey	1
15	Medical Board Australia and AHPRA (16)	Medical Training Survey 2022: Report for Aboriginal and Torres Strait Islander trainees	Australia	Cross-sectional survey	1
16	Milroy and Bandler (49)	Supervisor conversations: decolonisation in GP education	Australia	Discussion/conceptual	4
17	Milroy and Frayne (23)	Racism and cultural safety for Indigenous GP trainees	Australia	Qualitative/exploratory	3, 4
18	NACCHO (36)	Working Better for Medicare Review: Submission	Australia	Policy submission	1, 2, 3
19	Nychuk et al. (40)	Wise practices in Indigenous medical learner recruitment	Canada	Mixed-methods review	2, 3
20	RACGP (25)	Aboriginal and Torres Strait Islander Cultural and Health Training Framework	Australia	Policy framework	1, 2, 4
21	Reath et al. (38)	Supporting Aboriginal and Torres Strait Islander cultural educators and mentors in GP education	Australia	Mixed-methods study	2, 3, 4
22	Reid et al. (18)	AIDA Specialist Trainee Support Program Evaluation Report	Australia	Program evaluation	1, 2, 3
23	Rourke et al. (43)	From pipelines to pathways: Memorial University rural generalist model	Canada	Program description/evaluation	2, 3
24	Taylor et al. (32)	National Report on the 2025 GP National Registrar Survey	Australia	Cross-sectional survey	1
25	Taylor et al. (33)	National Report on the 2024 GP National Registrar Survey	Australia	Cross-sectional survey	1
26	Tipene-Leach et al. (45)	Cultural safety and the medical profession in NZ	New Zealand	Framework analysis	3, 4
27	Worley et al. (37)	Northern Territory Medical Program: growing our own	Australia	Program description	2, 3

### 3.2 Domain 1: Aboriginal and Torres Strait Islander doctor/trainee workforce data

#### 3.2.1 National workforce numbers

The Medical Training Survey (2025) provides national data of respondents representing Aboriginal and/or Torres Strait Islander doctors in *training* across all career stages. Nationally, only 118 Aboriginal and/or Torres Strait Islander doctors in training responded to the 2025 Medical Training Survey, compared to 191 respondents in 2022 (16, 17). Although these figures are survey respondents and not the total number of Aboriginal and/or Torres Strait Islander doctors in training nationally, these counts must be read against a marked fall in the overall Medical Training Survey response rate (from 56.6% of eligible trainees in 2022 to 36.7% in 2025) (16, 17). The change therefore reflects, at least in part, reduced survey participation rather than a measured decline in the number of Aboriginal and/or Torres Strait Islander doctors in training, and is best interpreted as an indicator of persistent under-representation relative to population parity. Within the 2025 respondents, the largest proportion of Aboriginal and/or Torres Strait Islander doctors training in General Practice was 25% (17). This was followed by Emergency Medicine (16%), Surgery (12%), Physician Adult Medicine including specialties (11%), and Psychiatry (8%) (17). The 2022 respondent cohort reported the highest proportion of Aboriginal and/or Torres Strait Islander doctors in General Practice (26%), followed by Emergency Medicine (14%), Surgery (13%), Physician Adult Medicine including specialties (12%), Psychiatry (8%), and Obstetrics and Gynaecology (6%) (16). With respect to geographic distribution, the 2022 respondent cohort was predominantly located in metropolitan areas (57%), followed by regional (33%) and rural areas (9%), with 2% not specifying their location (16). By 2025, metropolitan representation among respondents decreased to 44%, while regional and rural areas were 40 and 16%, respectively (17). These proportions are drawn from different denominators and markedly different response rates and should be interpreted with caution. In absolute respondent terms, the regional count did not increase (approximately 63 in 2022 vs. 47 in 2025) and the rural count changed only marginally (approximately 17 vs. 19), so the apparent proportional growth should not be read as evidence of improved workforce distribution. The 2025 survey further reported that 56% of Aboriginal and Torres Strait Islander respondents experienced and/or witnessed bullying, harassment, discrimination, and racism during their training, compared to a national average of 30%, while 38% reported direct experiences of racism (17).

#### 3.2.2 Longitudinal and fellowship-level data

Longitudinal data from the AIDA (31) Specialist Medical College Self-Assessment Report, contextualise these figures within a broader national trajectory. Between 2020 and 2021, the number of self-identified Aboriginal and Torres Strait Islander fellows across all specialist colleges rose from 108 to 150 (31). While this was an increase of more than 25% of Aboriginal and Torres Strait Islander doctors, it still represented less than 0.2% of Australia's total specialist medical workforce due to simultaneous growth in the overall workforce (31). During the same period, the number of Aboriginal and Torres Strait Islander trainees enrolled in specialist colleges remained relatively steady at approximately 187–188 (31). Within the GP training context, the RACGP reported 60 self-identified Aboriginal and Torres Strait Islander GPs in training and 124 Fellows in 2021 (31).

At the fellowship level, the RACGP Framework confirms that as of June 2024, Aboriginal and Torres Strait Islander trainees constituted 1.3% of RACGP training enrolments and 0.39% of total GP Fellows, against a population parity target of 3.8% (25). The AIDA Specialist Trainee Support Program Evaluation similarly reported that only 0.3% of all medical specialists identified as Aboriginal and/or Torres Strait Islander (18). The National GP Registrar Survey 2025 reported that Aboriginal and Torres Strait Islander registrars comprised 2.3% of survey respondents, and all Aboriginal and Torres Strait Islander respondents indicated membership in the IGPTN, while also reporting satisfaction with its support (32). The IGPTN (22) Annual Report 2024–2025 documents steady membership growth across all states and territories, and Queensland is noted to be among the largest cohorts. Moreover, IGPTN's biannual workshops and weekly online study groups were seen as the primary structured peer support setup available to Aboriginal and Torres Strait Islander GP and Rural Generalist trainees nationally (22).

In 2024, Taylor et al. (33) reported in the National GP Registrar Survey that Aboriginal and Torres Strait Islander registrars comprised 2.3% of the survey respondents across the AGPT, Rural Generalist Training Scheme (RGTS), and Remote Vocational Training Scheme (RVTS) programs combined. This number represents 97 registrars in a total training population of 3,870 (33). Similar to the outcome of 2025, approximately 75% of Aboriginal and Torres Strait Islander registrar respondents indicated membership with the IGPTN, and almost all who engaged (91%), reported 100% satisfaction with its support (33). Across the full registrar cohort, 88% had participated in Aboriginal and Torres Strait Islander cultural education since commencing GP training, and 93% indicated satisfaction with it (33). However, only 19% had accessed a cultural mentor or educator for guidance, despite 70% reporting that they knew how to do so (33). Across these national datasets, a consistent gap was evident. Queensland-specific, Aboriginal and Torres Strait Islander-identified workforce data disaggregated by career stage and geographic distribution were largely unavailable, such that national figures had to serve as proxies for the Queensland context. The near-absence of Queensland-disaggregated data is itself a substantive finding of this review.

### 3.3 Domain 2: programs and initiatives in Queensland and Australia

#### 3.3.1 AIDA specialist trainee support program

The AIDA Specialist Trainee Support Program (STSP) Evaluation Report (18) describes an Aboriginal and Torres Strait Islander-led specialist trainee support evaluation program in Australia. The program provides targeted mentoring, advocacy, and tailored support for Aboriginal and Torres Strait Islander non-GP specialist trainees, and documented a 142% increase in Aboriginal and Torres Strait Islander Fellows and trainees between 2020 and 2023. The Aboriginal and Torres Strait Islander governance model, with AIDA leading program design and delivery, is identified as a critical enabler of program legitimacy, cultural safety, and effectiveness, and it is framed as self-determination in practice (18). Key recommendations from the evaluation include securing long-term sustainable funding, expanding the scope into clinical and pre-vocational settings, establishing local network coordinator roles, and mandating in-person cultural safety training across all medical training environments. The AIDA 2021 self-assessment report further confirmed that the structural features of Aboriginal and Torres Strait Islander-led support, such as dedicated mentoring, scholarship provision, targeted selection, and committee governance with mandatory Aboriginal and Torres Strait Islander representation at the board level, remain inconsistently implemented across most specialist medical colleges (31).

#### 3.3.2 Remote vocational training scheme

The Remote Vocational Training Scheme (RVTS) provides a place-based vocational training pathway for doctors working in rural, remote, and Aboriginal Medical Service (AMS) settings (34). The pathway is designed to support doctors in their progression toward RACGP and/or ACRRM fellowship while remaining in the same community (34). The program operates under two streams: a Remote Stream targeting Modified Monash Model (MMM)4–7 locations, and an AMS Stream that allocates 10 of the program's 32 annual training places to Aboriginal Medical Services in MMM2–7 areas (34, 35). An independent evaluation conducted in 2023–2024 highlight a 78% program completion rate, with 49% of graduates still working in their training community 2 years after completion, and 88–100% participant satisfaction across various indicators (35). Participants worked a mean of 1.6 years in the same practice before joining the program and remained for a mean of 3.6 years during training. This resulted in a total mean of 5.2 years in the same practice location, which is approximately three times longer than the expected retention for doctors in areas of equivalent rurality (35). To reflect the generative sustainability of the model, approximately 30% of current supervisors are RVTS graduates (35). The RVTS delivers training through remote supervision and distance education by using adaptable technology to provide real-time support and virtual small-group learning, as well as providing twice-yearly in-person workshops where family attendance is funded by the program (34). NACCHO (36) reported a decline in GP registrars working in remote and rural areas from a peak of 61 in the Northern Territory in 2016 to fewer than 10 by 2023. Since 2013, over 80% of RVTS enrolments across both streams have been International Medical Graduates; Aboriginal and Torres Strait Islander doctors were not identified as a distinct group in the program's administrative dataset, which showcases a limitation in monitoring First Nations participation in the program (34).

#### 3.3.3 Place-based medical education

Although the primary focus of this review is postgraduation medical training, two place-based medical education initiatives are included for their direct relevance to the Aboriginal and Torres Strait Islander GP pipeline. These initiatives include the Northern Territory Medical Program (NTMP), which addresses the pre-graduation pipeline as a foundational context for workforce outcomes, and the General Practice Education and Training (GPET) Framework, which governs post-graduation vocational training in Aboriginal and Torres Strait Islander health.

The NTMP commenced in Darwin in 2011 as a distributed Flinders University medical program designed to develop the medical workforce for the NT (37). Selection prioritised Aboriginal and Torres Strait Islander NT residents first, followed by non-Indigenous NT residents, Aboriginal and Torres Strait Islander Australians outside the NT, and other Australian or New Zealand citizens. All students received fee-free, government-bonded places. A dedicated Aboriginal and Torres Strait Islander Entry Stream, which involves a third distinct pathway alongside standard graduate entry and a Bachelor of Clinical Science double degree, was established in collaboration with AIDA, to provide academic, pastoral, and financial support that includes an Elder on campus and dedicated Aboriginal and Torres Strait Islander staff (37). The Entry Stream selection process evolved iteratively over 4 years from an initial 2-week residential program to a more rigorous assessment that involve written examinations, oral presentations, project reports, and a 12-week online science program. According to Worley et al. (37), among the 144 students enrolled between 2011 and 2016, 20 were Aboriginal and Torres Strait Islander people, and 14 of those were NT residents, with seven having graduated by the time of reporting. Aboriginal and Torres Strait Islander students comprised 14% of entrants and graduates over this period. Worley et al. (37) note that this proportion, compares favourably to any other Australian medical school. Of the first graduating cohort of eight, 75% remained in the NT following the completion of their obligatory return-of-service period. Key lessons that Worley et al. (37) leveraged from the NTMP program include the importance of leadership at the highest institutional levels, accommodation of community and family pressures experienced by Aboriginal and Torres Strait Islander students, recalibration of selection processes iteratively, and the development of networks rather than operationalising what they termed as “hub-and-spoke” models of distributed training support.

Martin and Reath (4) foreground the historical development and evaluation of the GPET Framework, which required regional training providers to deliver Aboriginal and Torres Strait Islander health training in accordance with the RACGP and ACRRM curricula. The Framework was endorsed by the GPET Board in 2003, following curriculum mandates by the RACGP in 1997 and ACRRM accreditation. Training posts in Aboriginal Community Controlled Health Services (ACCHSs) were financially supported through a salary reimbursement program, with 84% of Aboriginal and Torres Strait Islander health training posts located in ACCHSs by 2005 (4). Evaluations of the Framework in 2005 and 2008 reported increases in Aboriginal and Torres Strait Islander training placements, although variations in implementation across regional training providers were noted. Both evaluations recommended enhanced recruitment, recognition, and support of Aboriginal and Torres Strait Islander educators and mentors, and improved tracking of training and related outcomes (4).

#### 3.3.4 Cultural educators and cultural mentors

Reath et al. (38) provide evidence within the Australian context on Cultural Educator and Cultural Mentor (CE/CM) model in GP training. The study distinguishes between cultural education, which provides baseline preparation for practice with Aboriginal and Torres Strait Islander patients, and cultural mentoring, characterised as longitudinal, relationship-based learning particularly valued by registrars working in Aboriginal and Torres Strait Islander communities. Factors identified as essential for sustaining CE/CM roles include adequate remuneration, formal employment structures, strong partnerships with medical educators, and defined career pathways (38). CE/CM roles are reported as under-resourced and inconsistently implemented, and cultural mentoring is frequently provided without pay or formal recognition (38).

The AIDA (31) self-assessment report identified CE/CM-equivalent roles across specialist medical colleges as among the most structurally underdeveloped. This is epitomised by the consistent absence of remuneration, career pathways, and formal recognition in those medical colleges (31). Fewer than half of the colleges reported meaningful progress in establishing or sustaining dedicated mentoring and support roles for Aboriginal and Torres Strait Islander trainees during the reporting period (31).

#### 3.3.5 RACGP Yagila Wadamba program

The RACGP's Yagila Wadamba Program (meaning learn to heal in Woi Wurrung language), was developed by the RACGP Aboriginal and Torres Strait Islander Health faculty (31). It is a two-day, biannual workshop planned with RACGP fellowship examination cycles, with primary focus on Applied Knowledge Test (AKT) and Key Feature Problem (KFP) exam preparation (31). The program is fully funded by the RACGP Aboriginal and Torres Strait Islander Health Faculty, covering flights, accommodation, and transfers for all participants (31). Priority access is given to Aboriginal and Torres Strait Islander registrars with upcoming fellowship examinations. The program also functions as a peer networking and mentoring opportunity, bringing registrars together with Aboriginal and Torres Strait Islander GP Fellows and medical educators (31). The AIDA (31) self-assessment report confirmed that the RACGP used the Yagila Wadamba Program to fund AIDA GP member attendance. No published evaluation data are available on the impact of Yagila Wadamba on fellowship examination pass rates, registrar completion, or post-fellowship workforce retention.

#### 3.3.6 Indigenous general practice trainee network (IGPTN)

The IGPTN (22) operates as a parallel peer support and education setup for Aboriginal and Torres Strait Islander GP and Rural Generalist trainees across both RACGP and ACRRM fellowship pathways. Originally operating under the auspices of General Practice Registrars Australia (GPRA), the IGPTN was incorporated as a self-determined Aboriginal and Torres Strait Islander corporation in late 2023 and entered a direct government grant agreement with the Department of Health, Disability and Ageing in July 2024 (22). In 2024–25, the IGPTN delivered two face-to-face workshops. The first workshop was held in October 2024 at Naarm (Melbourne), and the second workshop was held in May 2025 at Kaurna Country (Adelaide). These workshops were scheduled to align with college examination dates, and they integrated exam preparation, yarning circles, clinical skills development, peer mentoring, and cultural immersion (22). In addition to workshops, IGPTN delivers weekly online study groups, mock examinations, and one-on-one mentoring facilitated by Aboriginal and Torres Strait Islander GP and Rural Generalist Fellows (22).

The IGPTN Annual Report of 2024–2025 recorded a total revenue of $904,152 against a total expenditure of $678,809, with a total surplus of $225,343 (22). The three largest cost categories were employment ($325,691), workshop delivery ($128,614), and travel and accommodation ($110,050) (22). According to the report, membership grew steadily across all states and territories during the reporting period, and Queensland was among the largest state cohorts (22). As highlighted earlier, member satisfaction with the IGPTN's exam support was rated highly in the National GP Registrar Survey, and all Aboriginal and Torres Strait Islander registrar respondents reported membership to the IGPTN (22, 32). IGPTN identified ongoing challenges such as structural barriers to cultural safety in training environments, workload pressures on trainees, and funding limitations that constrains the scope and reach of program delivery (22).

#### 3.3.7 Joint colleges training services

The Joint Colleges Training Services (JCTS), established in 2022 as a joint venture between the ACRRM and the RACGP, represents the primary national setup for Aboriginal and Torres Strait Islander cultural education and mentorship within the AGPT program (39). Operating in every state and territory, JCTS delivers cultural education workshops for GP registrars and supervisors, cultural observation visits, cultural mentor workshops, practice manager workshops for ACCHSs, a Roving Registrar Program, and direct support for Aboriginal and Torres Strait Islander GP registrars (39). JCTS's (39) strategic plan articulates a vision of “strong communities and healthy futures through embedding Aboriginal and Torres Strait Islander ways in GP training” (p. 6). This strategic plan is structured across six themes, to wit: cultural safety and responsiveness, cultural education excellence, community focus, wellbeing at work, strong partnerships, and resource efficiency. The plan identifies the protection and preparation of Aboriginal and Torres Strait Islander registrars to thrive in mainstream practice placements; with the prospect of growing the Aboriginal and Torres Strait Islander cultural mentor workforce, as direct strategic priorities (39).

#### 3.3.8 The AGPT performance and outcomes framework

The AGPT Program Performance and Outcomes Framework 2026–2030 (27) provides a national accountability structure within which the JCTS operates. The Framework sets a target of Aboriginal and Torres Strait Islander registrar enrolment averaging 3% over a rolling three-year period, rising proportions of FTE core GP training weeks in ACCHSs and AMSs from 6% in 2026 to 8% by 2030, and 100% of both fellowing registrars and supervisors completing cultural education (27). The Framework assigns JCTS shared accountability with GP colleges for delivering cultural education, supporting cultural educators and mentors, and enabling the program's cultural safety objectives (27). The JCTS operates within a broader system that the Framework identifies as structurally under-resourced. It acknowledges that only 1% of doctors are Aboriginal and/or Torres Strait Islander against a 3.3% population share; and acknowledges that the use of health services by Aboriginal and Torres Strait Islander peoples increases when they receive culturally appropriate care (27).

### 3.4 Domain 3: international evidence from Canada and New Zealand

#### 3.4.1 Canada: admissions reform and pathways

Nychuk et al. (40) synthesise wise practices in Indigenous medical learner recruitment and admissions across Canadian medical schools. The review identified cultural safety assessment criteria embedded in admissions processes, Indigenous community involvement in candidate selection, transition support structures, and institutional accountability mechanisms (40). The review also noted that while most Canadian medical schools have developed some form of Indigenous-specific admissions process, support for Indigenous learners at the postgraduate level remains significantly less developed (40). Black et al. (41) however, found that 81% of Indigenous medical students reported that the availability of a dedicated Indigenous admissions pathway would influence their choice of residency program. Additionally, mentorship from Indigenous physicians was identified as the most important feature associated with the choice of admission. Significant barriers identified that would deter students include fear of stigma and concerns about being perceived as not having earned their position (41). Koea et al. (42), through a narrative review of affirmative action programmes internationally, identified that effective postgraduate pipeline intervention incorporates early engagement at the secondary school level, holistic selection, targeted bridging support, and sustained mentorship through to leadership appointment. Koea et al. (42) further noted that affirmative action programmes functioning as single-entry interventions rather than longitudinal pipeline commitments, risk the phenomenon of “pepper potting”, whereby isolated trainees face cultural assimilation rather than genuine inclusion.

#### 3.4.2 Canada: Memorial University and national workforce development

At the Memorial University of Newfoundland, Rourke et al. (43) described end-to-end pathways to rural practice model across four sequential stages: pre-admission outreach, undergraduate medical training, postgraduate Family Medicine residency, and post-fellowship professional development. The Aboriginal Initiative includes the MedQuest high school engagement program and an admissions process that explicitly considers geographic and minority representation among both selectors and candidates (43). There were 23 Memorial Doctor of Medicine (MD) students who self-identified as Aboriginal as of September 2016, and 91% of those students were from rural locations. Among MD graduating classes of 2011–2020, 56% of graduates spent the majority of their pre-18 years in rural locations. This demonstrates the program's deliberate rural-selection pipeline (43). Graduate outcomes are tracked longitudinally through the Learners and Locations database, which follows students from program entry through to practice location (43). Furthermore, rural immersion is embedded throughout the program. This was shown by how 89% of Year 3 Family Medicine placement weeks (graduating classes 2011–2018) took place in rural communities, and 100% of Family Medicine residents from graduating classes 2011–2013 completed some rural training, with an average of 52 rural training weeks out of a total average Family Medicine training time of 95 weeks (43). For the 2016–2017 1st-year residents, 53% of the 1st year was completed in rural locations. Notwithstanding the progress of the program, Rourke et al. (43) identified ongoing challenges that require continued collaboration with governments, medical associations, health authorities, and communities to achieve sustainable rural healthcare delivery.

Anderson and Lavallee (44) describe parallel national initiatives like the 2005 Kelowna Accord target aimed at doubling Indigenous physician numbers over 10 years, and this was supported by a C$100 million Aboriginal Health Human Resources Initiative. The Association of Faculties of Medicine of Canada (AFMC) and the Indigenous Physicians Association of Canada (IPAC) partnership established two interdependent workstreams, to wit, recruitment and retention, and curriculum reform. These workstreams were framed as necessary components of health system transformation (44).

#### 3.4.3 New Zealand: cultural safety and anti-racism

Tipene-Leach et al. (45) trace the evolution of cultural safety training in New Zealand from early knowledge-based teaching through skills-based cultural competency to self-reflective cultural safety practice; and this culminated in the Medical Council of New Zealand's 2019 Statement on Cultural Safety. Curtis et al. (46) proposed refined definitions that distinguish cultural safety from cultural competency. According to them, cultural safety is characterised by critical self-reflection on power and privilege, and determined by patients and communities rather than practitioners. However, cultural competency concerns awareness of diverse worldviews and the adaptation of practice. Curtis et al. (46) further note that both concepts are necessary and should not be conflated, nor substitute for the rights-based obligations owed to Indigenous peoples. Cormack et al. (47), in a national survey of Māori medical students and physicians, identified the impact of racism, discrimination, bullying, and harassment in causing many Māori physicians to consider leaving medicine.

### 3.5 Domain 4: barriers, enablers, and cultural safety frameworks

#### 3.5.1 Systemic racism and the dual burden of proof and responsibility

Milroy and Frayne (23) provide evidence of racism in GP training in Australia. They highlighted that Aboriginal and Torres Strait Islander GP trainees experience racism that operates at interpersonal, internalised, and systemic levels across their professional lives. Accordingly, they maintain that adaptive and maladaptive responses such as avoidance, internalised anger, and attempts to modify the self so that racism does not recur, were some of the measures these cohorts of trainees adopted to deal with racism; however, these adaptive and maladaptive responses carried a significant psychological cost over time (23). Burm et al. (48) report comparable dynamics among Indigenous medical learners in Canada. They identified two distinct burdens: the burden of proof, which requires continuous justification of academic fitness; and the burden of responsibility, which requires service as cultural educators, committee representatives, and Indigenous advocates. They noted that these responsibilities are frequently required of Indigenous medical learners without acknowledgement or compensation. Burm et al. (48) further identify institutional climate transformation, cluster hiring of Indigenous health expertise, and mandatory anti-racist and trauma-informed training for all staff, as necessary structural responses to ameliorate the impact of these burdens. As earlier highlighted, Cormack et al. (47) buttress this by identifying in a national survey of Māori medical students and physicians, that over 90% reported experience of direct racism, while over 60% had considered leaving medicine, and more than one-third had specifically contemplated a break due to racism, discrimination, and bullying.

The AIDA (31) self-assessment document reports that more than one-third of specialist medical colleges had made no progress on mandatory and assessable curriculum content on cultural safety and Aboriginal and Torres Strait Islander health, and that most cultural training resources offered across colleges remained non-mandatory, awareness-focused rather than safety-focused, and delivered online rather than through face-to-face engagement. The IGPTN Annual Report confirmed that Aboriginal and Torres Strait Islander GP trainees continue to identify structural barriers to cultural safety in training environments as one of their most significant ongoing challenges (22).

#### 3.5.2 Financial hardship, geographic dislocation, and inflexibility

Reid et al. (18) underscore that financial hardship, racism, inflexible training arrangements, and limited pathways are exacerbating barriers to the recruitment and retention of Aboriginal and Torres Strait Islander specialist trainees. Inflexible training arrangements creates structural mismatches for trainees managing familial, cultural, and community obligations alongside training requirements (18). Milroy and Frayne (23) emphasis how financial pressures interact with racism and the absence of cultural safety to produce severe challenges, including navigating Aboriginal and Torres Strait Islander patient advocacy and identity disclosure while maintaining professional practice.

The 2024 National GP Registrar Survey provides additional specificity on financial barriers, reporting that 55% of all GP registrars earned less in GP training than in their final year of prevocational hospital training, and 53% were also earning below $60,000 in Semester One of 2024 (33). The survey also found that 43% of registrars received no additional support payments during that period, and this was compounded by the financial gap that was most pronounced in the earliest stages of training (33). NACCHO (36) identified pay parity and entitlement portability for registrars transitioning from hospital to GP training as a structural barrier requiring government intervention. Additionally, NACCHO (36) reports from its sector census that 94.4% of Aboriginal Community Controlled Health Organisations (ACCHOs) face workforce recruitment challenges and 69.6% face retention challenges. At the same time, GPs were identified as the most difficult role to fill (36). NACCHO specifically recommends building ACCHO supervisory and training capacity as a structural strategy for sustaining GP training placements in First Nations community health settings (36).

#### 3.5.3 Enablers: what the evidence shows works

Indigenous-led peer networks are identified in multiple literature as among the most valued and protective elements of the training pipeline. All Aboriginal and Torres Strait Islander GP registrar respondents in the 2025 National Registrar Survey indicated membership in the IGPTN and reported satisfaction with its support (32). Reid et al. (18) report that AIDA STSP trainees described peer connection as essential to addressing the isolation inherent in being a minority trainee in a non-Indigenous institution. Burm et al. (48) similarly identify peer networks as providing a crucial sense of not being alone. The IGPTN Annual Report 2024–2025 foregrounds that biannual face-to-face workshops integrating exam preparation, yarning, mentoring, and cultural immersion are described by participants as being culturally safe and empowering (22). These are also the enabling features of the RACGP Yagila Wadamba exam preparation Program (31).

Targeted financial support was also identified as a consistent enabler. In this light, Reid et al. (18) reported that AIDA STSP financial assistance was consistently valued by trainees. The RACGP Framework commits to exploring scholarships, fee waivers, and emergency financial assistance as structural responses (25). The IGPTN Annual Report documented travel and accommodation expenditure of $110,050 in 2024–2025, with total program expenditure of $678,809 (22). The AIDA (31) report noted that most specialist college scholarship and bursary programs either remained static, were disrupted by COVID-19, or were insufficient in scope relative to the financial barriers experienced by Aboriginal and Torres Strait Islander trainees (31).

Furthermore, longitudinal cultural mentoring is identified by Reath et al. (38) as the form of support most valued by registrars, yet most structurally neglected in the literature. Reid et al. (18) note Aboriginal and Torres Strait Islander-led program design as a prerequisite for program legitimacy, cultural safety, and effectiveness. Holistic admissions processes that value lived experience, community service, and resilience alongside academic credentials are identified by Nychuk et al. (40), Black et al. (41), and Koea et al. (42) as important structural features of effective Indigenous medical learner recruitment. Milroy and Bandler (49) recognise supervisor role modelling and active bystander behaviour as practical operationalisations of institutional anti-racism in quotidian training. The AIDA (31) report confirms that colleges making the most measurable progress were those in which Aboriginal and Torres Strait Islander health and workforce priorities were actively championed at the highest levels of college leadership.

## 4 Discussion

Enhancing the pipeline of Aboriginal and Torres Strait Islander doctors into and through general practice training in Queensland is crucial to serve the health needs of underserved communities, which are predominantly Aboriginal and Torres Strait Islander people in Queensland. To this end, this scoping review mapped the evidence across 27 sources on the Aboriginal and Torres Strait Islander GP or Indigenous doctors workforce pipeline. Through our synthesis, four overarching themes emerged across the following domains: (i) workforce data confirming underrepresentation across all career stages and geographic maldistribution; (ii) promising but under-evaluated Australian programs; (iii) directly transferable lessons from Canada and New Zealand; and (iv) barriers, enablers, and cultural safety frameworks as prerequisites for pipeline success.

A recurring theme across the workforce-data domain was the scarcity of Queensland-specific, Aboriginal and Torres Strait Islander-identified evidence disaggregated by career stage and geography. This data gap constrains Queensland-level workforce planning and as noted below, points to a clear priority for future data collection. We therefore treat it not merely as a limitation of the present review but as a finding about the state of the evidence itself.

In drawing on the Canadian and New Zealand evidence, we note both its transferability and its limits. Several features support transferability to the Australian and Queensland context, such as comparable settler-colonial health systems, broadly similar Indigenous health inequities, English-language medical training, and the shared use of cultural-safety and pipeline or affirmative-action frameworks. At the same time, contextual differences temper direct translation, including differences in health-system financing and governance, the distinct treaty and rights context in New Zealand, the particular geographic and jurisdictional configuration of Australian general practice training, and the diversity of Aboriginal and Torres Strait Islander communities within Queensland. We therefore treat the international evidence as offering transferable lessons to be adapted to local Queensland governance and community structures, rather than as directly importable models.

### 4.1 Persistent underrepresentation and a participation gap

One of the central findings of this review is that Aboriginal and Torres Strait Islander participation in medical training remains below parity at every career stage. The number of Aboriginal and/or Torres Strait Islander doctors in training who responded to the Medical Training Survey fell from 191 in 2022 to 118 in 2025 (16, 17). However, the overall survey response rate also declined markedly over this period (from 56.6% of eligible trainees in 2022 to 36.7% in 2025) (16, 17). So this change is best understood as a reduction in the number of respondents rather than a measured decline in the number of Aboriginal and/or Torres Strait Islander doctors in training. Interpreted with that caution, it reinforces the broader pattern of underrepresentation that persists well below the national population parity target of 3.8%.

The longitudinal AIDA self-assessment data contextualises the declining figures by indicating that Aboriginal and Torres Strait Islander fellows across all specialist colleges increased by 25% between 2020 and 2021. Over the same period, the overall specialist workforce grew only modestly (by approximately 3%), and the share of specialists identifying as Aboriginal and Torres Strait Islander remained below 0.2% (31). The document further reported that the number of trainees across specialist colleges remained essentially flat at 187–188 across the same period (31). This confirms that fellowship growth was not being fed by a growing training cohort. In fact, it reveals that the modest gains at the entry or fellowship end do not translate into cumulative growth because attrition at intermediate transition points is persistent and unmeasured (18, 42). Apropos to the specialty distribution data, General Practice remained the dominant training specialty in both 2022 and 2025, because of how central it is in Aboriginal and Torres Strait Islander community health through the Aboriginal Community Controlled Health sector (4, 36).

### 4.2 Cultural unsafety as a driver of attrition

A consistent and cross-jurisdictionally validated finding of this review is that cultural unsafety in medical training environments is a structural driver of attrition that operates throughout the training process. Consequently, this factor disproportionately affects Aboriginal and Torres Strait Islander trainees at every stage. The finding that 56% of Aboriginal and Torres Strait Islander trainees experienced or witnessed bullying, harassment, discrimination, and racism in 2025, combined with 38% reporting direct racism (17), situates the training environment in Australia within a pattern that exacerbates the attrition rate. This trend is tantamount to what Cormack et al. (47) reported for Māori physicians in New Zealand, where over 90% reported direct racism, and over 60% had considered leaving medicine entirely. The convergence across jurisdictions is theoretically significant as it indicates that the experiences documented are not idiosyncratic local failures, but systemic features of medical culture in settler-colonial health systems. This finding is consistent with Milroy and Frayne's (23) characterisation of racism as operating simultaneously at the interpersonal, internalised, and systemic levels in Australian GP training.

Burm et al.'s (48) identification of the dual burden of proof and responsibility, provides a conceptual framework that is directly applicable to the Australian context. Coupled with the discrimination that Aboriginal and Torres Strait Islander trainees experience, they are simultaneously required to justify their academic legitimacy and carry the disproportionate cultural labor of being cultural mentors, representatives, advocates, and educators for their communities within institutions that have not structurally prepared them to share this load. These dual burdens of “proof” and “responsibility,” as Burm et al. (48) assert, are additional, unremunerated, and largely invisible workloads superimposed on the already demanding demands of postgraduate medical training, and they are concentrated in a population that is already numerically isolated.

The AIDA self-assessment data confirm that institutional preparations for these challenges remains inadequate. More than one-third of specialist colleges had made no progress on mandatory and assessable curriculum content on cultural safety and Aboriginal and Torres Strait Islander health, despite many being engaged in curriculum review processes for extended periods (31). Most cultural training resources across colleges remained non-mandatory, online, and awareness-focused rather than safety-focused, consistent with the literature that identifies these approaches as insufficient to produce behavioural change in clinical culture (45, 46). The JCTS (39), as the sole national body mandated to deliver cultural education across both GP colleges, represents the primary institutional apparatus through which the AGPT program attempts to operationalise cultural safety at scale. However, its strategic plan acknowledges that the preparation and protection of Aboriginal and Torres Strait Islander registrars in mainstream settings remains an ongoing priority rather than an achieved standard (27, 39). The distinction Curtis et al. (46) draw between cultural competency and cultural safety is critically important here because competency frameworks locate the problem in the knowledge deficits of individual practitioners, while safety frameworks locate it in the power relations and institutional structures that determine whether Indigenous people can be safe in health encounters. Awareness training only addresses the former while leaving the latter untouched.

Milroy and Bandler (49) identified supervisor role modelling and active bystander behaviour as the most proximate practical levers available to GP training programs. Supervisors who actively interrupt racist behaviour, name it, and model anti-racist practice in clinical settings create the micro-environments of safety that registrars require to complete their training (49). This implies that GP supervisors are the primary relational environment within which Aboriginal and Torres Strait Islander registrars develop as clinicians, and the quality of that environment determines whether registrars remain in training or exit. The systematic failure to equip supervisors with anti-racist tools, documented across both Australian GP training and comparable Canadian settings, further represents a structural deficit with direct implications for the participation gap and underrepresentation articulated in the workforce data.

### 4.3 Indigenous self-determination as non-negotiable

The third major finding of this review is that programs and initiatives in which Indigenous peoples hold genuine governance authority, not subjected to only advisory roles or cultural consultation, but decision-making power, produce qualitatively and quantitatively better outcomes than programs that are institutionally hosted with Indigenous participation.

The AIDA STSP, led by an Aboriginal and Torres Strait Islander organisation and evaluated independently by an Aboriginal and Torres Strait Islander consulting firm, documented a 142% increase in the number of fellows and trainees between 2020 and 2023 (18). The evaluation attributes this directly to the Aboriginal and Torres Strait Islander governance model, which enabled the program design to be genuinely responsive to trainee needs, culturally safe in its operation, and credible in its advocacy with colleges (18). The IGPTN's achievement of independent incorporation in 2023 and direct government grant-holding in 2024 similarly represents the operationalisation of self-determination that has produced near-universal uptake among Aboriginal and Torres Strait Islander GP registrars, with a 100% membership rate among survey respondents, and high satisfaction ratings (22, 32). These outcomes reflect what Saunders et al. (50) describe as the indispensable value of First Nations-led governance, which showcases a structural condition for program legitimacy and effectiveness.

The JCTS presents a more complex governance in the case; because while it is being led by an independent Aboriginal and Torres Strait Islander Governing Board Chair and operationally committed to cultural integrity, it is a joint venture of ACRRM and RACGP, and its board membership is predominantly drawn from college executives (39). The degree to which this governance model enables Aboriginal and Torres Strait Islander-led decision-making, as distinct from Aboriginal and Torres Strait Islander-informed delivery, warrants critical scrutiny in light of the evidence favouring full self-determination. The AGPT Framework's assignment of JCTS as jointly accountable for cultural safety outcomes, without specifying Aboriginal and Torres Strait Islander community governance requirements, reflects this ambiguity at the national policy level (27).

Evidence from Canada highlights the AFMC–IPAC partnership, in which the Indigenous Physicians Association of Canada co-led the two interdependent workstreams of recruitment and curriculum reform, representing a qualitative shift from institutional goodwill to shared governance (44). Memorial University's Aboriginal Initiative embedded Indigenous community involvement in admissions, curriculum development, and student support (43). As of September 2016, 23 Memorial MD students self-identified as Aboriginal, and of the 22 with recorded background locations, 91% came from rural areas. Memorial also reports a rural practice rate of 26.9% among its Family Medicine postgraduates 2 years after training, against a national average of 13.3%. Nychuk et al.'s (40) synthesis of wise practices across Canadian medical schools similarly identifies Indigenous community involvement in candidate selection as among the most consistently identified features of effective Indigenous-specific admissions processes.

These findings have direct implications for Queensland. IGPTN's (22) identification of Queensland as among its largest state membership cohorts, suggests that an existing Aboriginal and Torres Strait Islander-led setup with genuine Queensland reach is already in place and functioning. The strategic challenge is to leverage and resource this setup, and lean on what they can inform in comparable contexts.

### 4.4 The financing gap

A finding that cuts across every program included in this review is that financial instability, whether through annual funding cycles, insufficient scope, or inadequate remuneration of cultural roles, represents a structural barrier to pipeline sustainability that cannot be resolved by program design alone. It requires sustained policy commitment and commensurate resourcing. According to the financial data in the 2024–2025 Annual Report of IGPTN (22), travel and accommodation alone consumed $110,050 of their expenditure. This reflects the geographic reality of a training cohort increasingly distributed across regional and rural settings, thereby, requiring financial assistance for conference or training attendance. A network operating at this scale on a single annual government grant is structurally fragile, and the IGPTN itself identifies funding limitations as one of its most significant ongoing constraints (22). The AIDA STSP Evaluation makes the same point, as Reid et al., (18) argue that annual funding blocks, prevent strategic planning, create staff burnout, and generate program instability that flows directly to trainees.

The RACGP (25) Framework's commitment to exploring scholarships, fee waivers, and emergency financial assistance is an important acknowledgement of the financial barriers to training, but the language of exploration is not equivalent to structural commitment. AIDA (31) data confirm that most specialist college scholarship and bursary programs remain static or insufficient. This was compounded by the disruptions caused by COVID-19 and its aftermath, which disproportionately affected the financial support available to Aboriginal and Torres Strait Islander trainees precisely when the burden of training was highest (31). Reath et al. (38) further report that cultural mentors who provide some of the most valued and impactful support in GP training, routinely provide this work without pay or formal recognition, and thus, renders the arrangement as somewhat exploitative and unsustainable. The escalating full-time equivalent (FTE) targets of the AGPT Framework for ACCHSs and AMSs placements, going from 6% in 2026 to 8% by 2030, will increase demand on JCTS and CE/CM setup precisely as the evidence confirms that these roles are most structurally under-resourced (27, 38).

Geographically, the rise in the proportion of Aboriginal and Torres Strait Islander respondents based in regional (33%−40%) and rural (9%−16%) settings between 2022 and 2025 might suggest a move toward better-distributed training, but this reading does not hold once the sharply lower response rates are accounted for (16, 17). The underlying respondent numbers were, if anything, flat to declining (regional approximately 63 down to 47; rural approximately 17–9), so the shift is a feature of who answered the survey rather than measured workforce growth (16, 17). Nonetheless, it also means that a growing proportion of this already thin cohort is training in settings where supervision setup, professional development access, and cultural support are limited and costly to deliver. Without dedicated investment in supervision capacity, CE/CM role development, and travel support in these settings, the geographic shift risks producing additional attrition rather than improving service to underserved communities.

The financing gap extends beyond program funding to the remuneration structure of general practice training. The 2024 National GP Registrar Survey found that 55% of all GP registrars earned less during training than in their final prevocational year, and 43% received no additional support payments in Semester One of 2024 (33). For Aboriginal and Torres Strait Islander doctors carrying greater financial obligations through their training pathways, this structural pay gap at the hospital-to-GP transition intensifies the risks of making general practice an economically untenable specialty choice; precisely at the moment when commitment to the pathway is most critical.

### 4.5 The pipeline requires a longitudinal structure for intervention

The evidence consistently points that no single intervention is sufficient to address the structural inequities operating across the Aboriginal and Torres Strait Islander GP training pipeline. Instead, interventions need to be a longitudinal, coordinated pipeline structure that begins before medical school, extends through every transition point to fellowship and post-fellowship practice, and is sustained by long-term, adequately resourced investment in Indigenous-led infrastructure. Koea et al.'s (42) framework for affirmative action programmes provides a useful scaffold for this setup. Their proposed pipeline from secondary school outreach through to faculty and leadership appointments, precisely maps onto the gaps visible in the Australian evidence. Australia has pre-graduation models in the NTMP that demonstrate promising outcomes for Aboriginal and Torres Strait Islander medical students, but these are geographically limited and lack a systematic national equivalent. Australia has postgraduation supports in the IGPTN, JCTS, and the RACGP's Yagila Wadamba that demonstrably serve trainees (31, 51), but lack adequate resourcing and longitudinal evaluation.

The end-to-end model of Memorial University offers an example of what this architecture looks like in practice (43). Its four-stage pipeline, starting from pre-admission outreach, undergraduate immersion, postgraduate rural residency, to post-fellowship professional development, tracked through a longitudinal database, produced rural practice rates roughly doubled the national average (26.9 vs. 13.3% 2 years post-training), sustained rural immersion across every training stage, and dedicated Aboriginal admission pathways. The key structural features include holistic selection, geographic embedding, longitudinal tracking, community engagement in governance, and sustained investment across all stages. These features are transferable to Queensland, although adaptation to local Aboriginal and Torres Strait Islander community governance structures and the distinct landscape of Queensland's training regions would be essential.

For Queensland, the RVTS AMS Stream represents an existing structural mechanism for place-based GP training in First Nations community settings, but its failure to capture Aboriginal and Torres Strait Islander participation data and its predominant enrolment of International Medical Graduates limits its contribution to the Aboriginal and Torres Strait Islander pipeline (34). A dedicated Aboriginal and Torres Strait Islander stream within the RVTS, with active recruitment partnerships with IGPTN, AIDA, and NACCHO affiliates, and with Aboriginal and Torres Strait Islander identification captured systematically in administrative data, would convert an existing structural apparatus into a genuine pipeline contribution.

### 4.6 Implications for policy and practice

The findings of this review have six specific implications for Queensland and the broader national policy landscape, all oriented toward increasing the number of Aboriginal and Torres Strait Islander doctors who enter, progress through, and complete general practice training.

First, targeted recruitment must be prioritised alongside retention. The evidence confirms that recruitment gains are achievable when adequately monitored and resourced, as demonstrated by the RACGP's successful meeting of its government-set Aboriginal and Torres Strait Islander trainee acceptance targets in 2020 and 2021 (31). However, the national shortfall of respondents in Aboriginal and Torres Strait Islander doctor training numbers from 191 in 2022 to 118 in 2025 (16, 17) confirms that entry-point gains are not translating into pipeline sustainability. The RACGP could adopt holistic admissions processes, set and publicly report fellowship parity targets, and actively promote general practice as a career pathway for Aboriginal and Torres Strait Islander graduates in partnership with universities and Aboriginal community-controlled health organisations. Queensland Health could adapt the Queensland Rural Generalist Pathway to include specific Aboriginal and Torres Strait Islander recruitment targets and community-embedded training. The Australian Government has recently committed to demand-driven Commonwealth Supported Places specifically for First Nations students to study medicine, increasing the number of additional places per year to 150 by 2028 (20). Queensland Health, universities, and the RACGP should actively align their downstream recruitment and retention strategies with this upstream investment to ensure that this expanded cohort is supported through to GP fellowship.

Second, the monitoring and evaluation framework must be strengthened. Without longitudinal Aboriginal and Torres Strait Islander-identified workforce data from pre-vocational training through to post-fellowship practice locations, it is impossible to measure whether interventions are working. Queensland Health could advocate for mandatory Aboriginal and Torres Strait Islander identification fields across all GP training administrative datasets, linked to longitudinal tracking and governed by Aboriginal and Torres Strait Islander data sovereignty principles (36), consistent with the approach of Memorial University's Learners and Locations database, as well as the National Aboriginal and Torres Strait Islander Health Workforce Strategic Framework and Implementation Plan, which calls for information and data to be shared across systems to support health workforce planning, monitoring, and evaluation (26).

Third, cultural safety should be operationalised as a structural requirement, not just as a curriculum option. The evidence in the literature is pronounced that non-mandatory, online, awareness-focused cultural training, does not produce the institutional transformation required to retain Aboriginal and Torres Strait Islander trainees. While acknowledging the difficulty of meeting face-to-face always, the RACGP, ACRRM, and their relevant organisations should encourage face-to-face, in-person cultural safety training for all supervisors and medical educators. This should not be done as a one-off compliance requirement, but as an ongoing professional development standard with assessable components, as recommended by Reid et al. (18) and consistent with the cultural safety definition adopted by the Australian Health Practitioner Regulation Agency (52) and the RACGP (25) in their Aboriginal and Torres Strait Islander Cultural and Health Training Framework. Significant structural investment has already begun through the establishment of the JCTS in 2023, and the recommendations here are intended to build upon, rather than overlook these investments. GP supervisors, who constitute the primary relational environment for registrars, ought to be equipped and accountable as active anti-racism practitioners. Structures like the JCTS represent a promising foundation upon which more systematic accountability and mentoring frameworks can be developed. The RACGP's (53) vision for reconciliation calls for a profession free from racism, where all GPs provide culturally safe healthcare grounded in mutual respect and trust. Realizing this vision will require both the structural reforms now underway and the sustained professional development standards necessary to embed cultural safety as a living practice rather than a compliance exercise.

Fourth, Aboriginal and Torres Strait Islander-led infrastructure needs to be recognised as foundational rather than as an add-on. These infrastructures, such as the IGPTN, JCTS, and AIDA STSP, as well as other Aboriginal and Torres Strait Islander initiatives, especially within organisations such as those under the auspices of the RACGP, like the Yagila Wadamba Program (31, 51), are not add-ons to the mainstream training system. Rather, they are the primary cultural safety and peer support infrastructure that makes mainstream training survivable for Aboriginal and Torres Strait Islander trainees. Their funding should reflect a multi-year, indexed funding agreements, therein, replacing the annual grant cycles that such organisations identify as creating instability. This would enable strategic planning, staff retention, and program expansion into the pre-vocational and post-fellowship stages, where the evidence identifies the greatest gaps. Queensland's status as one of the IGPTN's (22) largest state cohorts, and JCTS's statutory mandate to deliver cultural education to 100% of AGPT registrars and supervisors, as set by the AGPT Framework (27, 39), positions it and other infrastructure/initiatives as indispensable to pipeline sustainability. Thus, it makes the case for Queensland Health and primary health networks to fund such infrastructure and other Aboriginal and Torres Strait Islander initiatives within the RACGP.

Fifth, a Queensland-specific Aboriginal and Torres Strait Islander GP pipeline strategy is warranted. The RACGP (25) Framework provides national policy direction, but fails to address the specific geographic, demographic, and service delivery challenges of Queensland. A state-level strategy, developed in partnership with IGPTN, NACCHO Queensland affiliate, AIDA, Aboriginal and Torres Strait Islander community-controlled health organisations, and Queensland university medical schools, should articulate a coordinated pipeline from secondary school engagement through to rural and remote GP fellowship, with explicit targets, a longitudinal tracking mechanism, and adequate resourcing across every stage.

Sixth, the financial disincentive structurally embedded in the transition from hospital to general practice training is likely to influence specialty choice, and disproportionately burdens Aboriginal and Torres Strait Islander doctors. General practice has long struggled to compete with other specialties on remuneration, and evidence confirms that this is not merely a matter of perception. The 2024 National GP Registrar Survey found that 55% of all GP registrars earned less in GP training than in their final year of prevocational hospital training, with 53% earning below $60,000 in Semester One of 2024 and 43% receiving no additional support payments during that period (33). That the pay gap is most pronounced at the earliest stages of training means that doctors are confronting the sharpest financial penalty precisely at the point when they are making or reconsidering their specialty commitment. For doctors weighing general practice against hospital-based specialties, this structural disparity sends an unambiguous signal regarding how the system values the work. For Aboriginal and Torres Strait Islander doctors, this signal is exacerbated by the financial realities that disproportionately accompany their pathways into medicine, including the likelihood of family financial obligations, geographic relocation costs, the burden of proof and responsibility, and a longer road through study without equivalent generational wealth to absorb income volatility. NACCHO (36) identifies pay parity and entitlement portability at the hospital-to-GP transition as structural barriers that requires government intervention. Its sector census data sharpens the urgency of this finding as 94.4% of ACCHOs face workforce recruitment challenges, 69.6% face retention challenges, and GPs are identified as the most difficult role to fill across the entire sector (36). The workforce most needed to serve Aboriginal and Torres Strait Islander communities is the one the system is most effectively pricing out of reach. Queensland Health, in partnership with the Australian Government and the RACGP, should pursue remuneration reform to close the prevocational-to-vocational pay gap. This should be aimed at targeted supplementary payments for Aboriginal and Torres Strait Islander registrars undertaking placements in Aboriginal community-controlled health organisations and rural or remote settings. Without addressing the financial structure that makes general practice a less rational economic choice, upstream investments in recruitment and cultural safety risks being systematically undermined at the point of specialty selection.

### 4.7 Strengths and limitations

The primary strength of this scoping review is that it identifies, describes, and synthesises existing evidence on strategies, programs, and obstacles relevant to strengthening the pathway of Aboriginal and Torres Strait Islander doctors into and through general practice training in Queensland, Australia.

However, this scoping review has some limitations. First, the evidence base is limited, and this reflects a genuine paucity of empirical research on this topic, particularly in Queensland. Most Australian program data in the included literature were descriptive or evaluative rather than rigorously comparative, thereby, constraining causal inference. Queensland-specific workforce data disaggregated by career stage, geographic distribution, and often lacking indications of indigeneity, are largely absent from the published literature. This warrants reliance on national data as proxies for Queensland-specific analysis.

Second, despite comprehensive databases and targeted grey literature searches, some relevant organisational reports, program evaluations, or annual updates may have been missed. This reflects an inherent limitation of grey literature searching in a field where significant program knowledge and operational data are held within organisations and are disseminated outside indexed databases. Additionally, publication bias may have influenced the evidence base, as programs with positive outcomes are more likely to be published or made publicly available, meaning that discontinued, failed, or underperforming initiatives may be underrepresented.

Limitations also attach to the construction of the search itself. The free-text term sets applied across databases were not fully harmonised, having been developed and refined platform by platform in light of retrieval. While a consistent conceptual structure was maintained throughout, this variation may have affected sensitivity unevenly across databases. Google Scholar also contributed the largest single share of records, and its limitations as a search source are acknowledged: it is not systematically indexed, it ranks results by relevance, it does not support field truncation in the manner of bibliographic databases, and it does not return fully reproducible result sets. Google Scholar was therefore used as a supplementary source, and the records retrieved were screened against the same eligibility criteria as all other records; nonetheless, the non-reproducibility of the tool is a limitation of the search.

A separate limitation concerns the provenance of the outcome data. A number of the outcome claims synthesised across the programs and initiatives domains (for example, program completion, retention, and satisfaction figures reported by IGPTN, AIDA and its Specialist Trainee Support Program, the RVTS, JCTS, and the RACGP) derive from documents produced by the organisations that deliver or evaluate the programs in question. Such self-reported organisational data carry an inherent risk of favourable reporting bias, and independent evaluation was available for only a subset of programs. These figures should therefore be interpreted as indicative rather than independently verified. This partly reflects the dearth of empirical literature on the topic, which meant that organisational reports and documents were frequently the principal sources of information available.

Third, the exclusion of non-English publications may have also led to some literature being uncaptured, but this was intentional given that the focus countries are Australia, Canada, and New Zealand, which have English as their primary language. Similarly, restricting the search to literature published from January 2016 onwards may have omitted relevant historical evidence, though this decision was made to ensure contemporary relevance. Consistent with the JBI scoping review methodology (28), a formal quality appraisal of individual sources was not conducted, as the aim of this review was to map the breadth of available evidence rather than to assess the certainty of effect estimates.

A further limitation concerns the screening process. Screening of each literature type, *to wit*, peer-reviewed articles and grey literature, was undertaken by the first and second authors respectively, rather than through full independent dual-screening of all records. To check consistency, each author independently cross-checked at least 20% of the records screened by the other, and any disagreements were adjudicated by the third and fourth authors. Agreement between the two screening authors was reached on all but one record, which was resolved by consensus of the full author team. A formal inter-rater agreement statistic (percentage agreement or Cohen's κ) was not calculated, however, as reconciliation was conducted collaboratively during team meetings. This staged screening-plus-cross-check approach is consistent with JBI scoping-review guidance but does not constitute full dual-screening.

Finally, the evolving policies and training environments present an additional limitation. The general practice training landscape has changed considerably across the 2007–2026 publication window, and some older literature may not fully reflect the current policy and operational context within which Aboriginal and Torres Strait Islander general practice registrars are trained. As new structures mature, the implications of some findings may also shift accordingly.

### 4.8 Directions for future research

The evidence gaps identified in this review are informative, as they provide directions for future research. These directives include prioritizing longitudinal cohort studies tracking Aboriginal and Torres Strait Islander medical graduates from qualification through to long-term practice location and specialty; rigorous evaluation of the Yagila Wadamba Program's impact on fellowship examination outcomes and post-fellowship workforce retention to encourage sustained funding and operationalisation; qualitative research with Aboriginal and Torres Strait Islander GP registrars in Queensland, including those in rural and remote settings, on their experiences of training environments, cultural education and safety, and support structure; economic analysis of the return on investment from Aboriginal and Torres Strait Islander GP pipeline programs relative to the health system costs of continued workforce inequity; evaluation of JCTS programs; and participatory action research co-designed with Aboriginal and Torres Strait Islander communities on the health system responsiveness gains attributable to having Aboriginal and Torres Strait Islander GPs practicing within community.

## 5 Conclusion

Our scoping review set out to map and synthesise existing evidence on strategies, programs, and frameworks relevant to strengthening the pipeline of Aboriginal and Torres Strait Islander doctors into and through general practice training in Queensland and Australia. Drawing on 27 sources ranging from peer-reviewed literature, government reports, program evaluations, to international comparators, the review has accomplished this through four domains of analysis namely, (i) workforce data; (ii) programs and initiatives; (iii) international evidence; and (iv) barriers, enablers, and cultural safety frameworks. The findings revealed that the Aboriginal and Torres Strait Islander medical training pipeline is underrepresented. This is set against the backdrop of growing policy commitment and mounting evidence of the direct link between Aboriginal and Torres Strait Islander health professionals and improved community health outcomes. In Queensland, a jurisdiction where Aboriginal and Torres Strait Islander populations are distributed across some of Australia's most geographically isolated and medically underserved communities, having a very few pre-fellowship Aboriginal and Torres Strait Islander GP registrars across the entire state paints a bleak picture.

The synthesis of our findings revealed the following: First, cultural unsafety is real within the context of Aboriginal and Torres Strait Islander medical training, and in extrapolation, drives attrition. The finding that 56% of Aboriginal and Torres Strait Islander trainees experienced or witnessed racism, bullying, or harassment in 2025 reflects a workforce policy concern with direct consequences for the sustainability of the pipeline. The RACGP (54), through its standards for general practice are committed to ensuring that Aboriginal and Torres Strait Islander health and cultural safety are integrated into its training program; because no recruitment strategy can outpace an environment that drives trainees out. Second, Indigenous self-determination in program governance is an evidence-based design requirement. Programs in which Aboriginal and Torres Strait Islander peoples hold genuine decision-making authority, rather than advisory roles, consistently produce better outcomes. Third, the financial disincentive embedded in the hospital-to-general practice transition risks undermining every other investments in the pipeline. When 55% of GP registrars earn less during training than in their final prevocational year, and general practice continues to lag behind other specialties in remuneration, the system sends a signal about how it values this work. Recruitment efforts, cultural safety programs, and mentorship initiatives can all be executed well, but if general practice still pays significantly less than other specialty pathways, many Aboriginal and Torres Strait Islander doctors are likely, when facing this transition, to be influenced in their specialty choice by financial disincentives. Therefore, remuneration reform, pay parity at the hospital-to-GP transition, and targeted financial incentives for placements in Aboriginal community-controlled health organisations and rural or remote settings, are encouraged as prerequisites for pipeline sustainability. Fourth, the financial structure sustaining Aboriginal and Torres Strait Islander-led infrastructure is inadequate. Annual grant cycles, unremunerated cultural labour, and the absence of multi-year indexed funding agreements constrain planning, accelerate staff burnout, and therefore, destabilise the very programs the evidence most strongly endorses. The Yagila Wadamba Program, IGPTN, and JCTS represent the most direct, evidence-aligned investments available for improving fellowship examination outcomes, cultural safety in training, and pipeline sustainability for Aboriginal and Torres Strait Islander GP registrars. Scaling these programs, alongside mandating culturally safe training environments, strengthening longitudinal data setup, and developing a Queensland-specific pipeline strategy with explicit workforce targets, constitutes the evidence base for action that this review has produced.

## Data Availability

The original contributions presented in the study are included in the article/Supplementary material, further inquiries can be directed to the corresponding author.
