# Supplementary material for: Strengthening the pipeline of Aboriginal and Torres Strait Islander doctors into and through general practice training in Queensland, Australia: a scoping review
Source: Front Med (Lausanne). 2026 Aug 24;13:1921854. doi: 10.3389/fmed.2026.1921854 (PMC13584867; doi:10.3389/fmed.2026.1921854)
Supplement: Supplementary file 1 [file Data_Sheet_1.pdf]

Supplementary Material

Charting Form

Author(s) Year Country	Population	Topic Domain	Intervention/ Program	Outcomes	Key Findings	Limitations	Relevance to Queensland
Anderson & Lavallee (2007) Canada	First Nations, Inuit, and Métis peoples of Canada; medical students and physicians; Canadian medical schools and national health organisations	Indigenous medical workforce; physician under-representation; recruitment & retention; cultural safety curriculum	National Aboriginal Health Human Resources Initiative (C$100M/5yr); AFMC–IPAC Aboriginal Health Task Group; university Indigenous admissions programs; scholarships; Role Model Program	Strategic plans for increasing Indigenous physicians; national Aboriginal health curriculum framework for all 17 Canadian medical schools; scholarships & recruitment programs	Indigenous people >4% of Canadian population but <0.25% of physicians; 2005 Kelowna Accord targeted doubling Indigenous physician numbers in 10 years; three focus areas: workforce numbers, cultural competency curriculum, community retention	No primary research data; estimates of Indigenous physician numbers were imprecise (100–150); significant gaps in health data for non-registered First Nations, Métis, and Inuit populations hampered workforce planning; initiatives described were aspirational at time of publication with limited evaluation evidence	Canadian context provides comparable international evidence on the scale of Indigenous physician under-representation and national policy approaches to workforce pipeline development, relevant to informing Queensland and Australian strategy
Australian Indigenous Doctors' Association (AIDA) (2021) Australia	Aboriginal and Torres Strait Islander specialist medical trainees, fellows, and applicants across 15 Australian specialist medical colleges; includes bi-national colleges covering Aotearoa New Zealand	Aboriginal and Torres Strait Islander specialist medical workforce; cultural safety training; recruitment and retention; mentoring; curriculum; reconciliation action planning; college governance	15 minimum and best-practice standards for specialist medical colleges developed with AIDA and the Council of Presidents of Medical Colleges (CPMC) in 2019, targeting recruitment, retention, and cultural safety for Aboriginal and Torres Strait Islander specialist trainees	College self-assessed progress against each of the 15 standards; number of self-identified Aboriginal and Torres Strait Islander applicants, trainees, and fellows across colleges; progress on RAPs, health committees, curriculum reform, scholarships, mentoring, cultural safety training, and advocacy	150 self-identified Aboriginal and Torres Strait Islander fellows across all colleges in March 2021 (up from 108 in March 2020, a rise of more than 25%); 187–188 Aboriginal and Torres Strait Islander trainees (relatively stable); Aboriginal and Torres Strait Islander doctors represented less than 0.2% of Australia's medical specialists; two-thirds of colleges progressed at least one-third of the 15 standards; four colleges progressed 8 or 9 standards; fewer than half of colleges progressed more than half of the standards; areas of strongest progress included RAP development, data reporting, website visibility, and adoption of the AHPRA cultural safety definition; areas of weakest progress included mandatory and assessable curriculum content on cultural safety, dedicated training positions, mentoring, and ongoing mandatory cultural safety training; more than one-third of colleges made no progress on mandatory and assessable curriculum content on Aboriginal and	Self-assessed data subject to social desirability bias; COVID-19 pandemic substantially impacted implementation of standards across the reporting period; progress not directly comparable across colleges due to different starting points, resourcing, and staff levels; report does not assess actual health outcomes for Aboriginal and Torres Strait Islander patients; data on trainee numbers rely on voluntary self-identification	Provides national cross-college benchmarking data directly relevant to the RACGP and ACRRM training pathways through which Queensland Aboriginal and Torres Strait Islander GP registrars train; findings on curriculum gaps, mentoring deficits, and cultural unsafety in specialist training environments corroborate evidence on barriers to fellowship attainment for Aboriginal and Torres Strait Islander GPs in Queensland; the Yagila Wadamba program referenced under RACGP is a Queensland-relevant initiative

					Torres Strait Islander health and cultural safety		
Black et al. (2023) Canada	Self-identified Indigenous medical students enrolled at any Canadian medical school; n=36 respondents (estimated 30% response rate); 58.3% Métis, 25% First Nations (status), 16.7% First Nations (non-status); predominantly pre-clerkship (61%), Western Canada (75%)	Postgraduate admissions pathways; residency program selection factors; mentorship; cultural safety in training; Indigenous health workforce pipeline	Indigenous Admissions Pathway (IAP): A parallel postgraduate residency admissions pathway developed by the University of Alberta Obstetrics and Gynaecology program, co-designed with Indigenous trainees, faculty, Elders, and community members	Acceptability of an IAP; factors influencing residency program choice; thematic analysis of barriers and facilitators to applying through an IAP; perceived impact of IAP on the healthcare system	81% of participants felt availability of an IAP would influence their residency program choice; 75% would apply through an IAP; location and proximity to family/support were the most important residency selection factors; mentorship from Indigenous physicians and community involvement were key desired features; eight themes identified including community engagement, cultural education, mentorship, and program commitment to Indigenous trainees; fear of judgement/stigma (14%), concern about entrance requirements, and program logistics identified as barriers; all participants believed IAPs would positively impact the healthcare system through increased Indigenous representation and culturally safe care	Small sample size; risk of selection bias; bot-related data contamination on social media distribution required response removal; students concerned about discrimination may not have participated; results not generalisable to all Indigenous trainees; geographic over-representation of Western Canada	Canadian context provides transferable evidence on IAP acceptability, stigma barriers, and the importance of mentorship and community engagement that informs postgraduate Aboriginal and Torres Strait Islander workforce pipeline strategies relevant to Queensland
Burm et al. (2023) Canada	Self-identified Indigenous medical learners (medical students and residents) and one recently graduated physician (within 12 months of graduation) from a single Canadian medical school; n=5; recruited via email, social media, and snowball sampling	Indigenous medical workforce development; identity and professional identity formation; racism in medical education; burden of proof and responsibility; mentorship; recruitment and retention; cultural safety in training	Not applicable	Narrative themes across three key transition points: preparing for and applying to medical school, completing undergraduate medical training, and determining specialty choice; two central burden constructs, namely burden of proof and burden of responsibility, and the role of connection and belonging in sustaining learners	Motivations for pursuing medicine were strongly tied to Indigenous identity, desire for social impact, and community obligations; applying to medical school created a 'burden of proof' where participants faced financial stressors, repeated demands to prove Indigenous ancestry, and stigma from peers who questioned their academic merit due to Indigenous admission streams; advancing through training created a 'burden of responsibility' where participants were disproportionately called upon without compensation to develop Indigenous health curricula, serve on committees, and correct racist content in teaching; participants found belonging and resilience through Indigenous peer networks, Indigenous physicians and mentors, cultural practices, and connection to land; institutional commitments to reconciliation were not matched by meaningful action to disrupt racism or reduce learner burden	Very small sample (n=5) from a single anonymised institution, limiting generalisability; participants' experiences are representative of Canadian context and should not be applied to other countries; voluntary self-identification means some Indigenous learners may not have been captured; those who had left medicine could not participate, potentially underestimating the burden experienced	Canadian context is highly transferable evidence on the lived experiences of Indigenous medical learners navigating admissions pathways, racism, identity tensions, and cultural taxation; directly informs Queensland strategies for Indigenous GP trainee recruitment, retention, mentorship, and institutional responsibility for reducing burden on Indigenous trainees

Cormack et al. (2024) New Zealand	Self-identified Māori medical students (years 2–6) and Māori physicians with a current annual practicing certificate in New Zealand; n=205 students (response rate 41.8%), n=200 physicians (response rate 23.4%); median age 23.1 years (students) and 36.6 years (physicians); majority women (67.2% students, 62.8% physicians)	Racism; discrimination; bullying; harassment; workforce retention; Indigenous medical education; patient safety; cultural safety	Not applicable	Prevalence of direct and witnessed racism, discrimination, bullying, and harassment ever and in the last 12 months; exposure to negative stereotypes and comments; witnessed discriminatory treatment of Māori patients; consideration of leaving or taking a break from medicine	Direct and witnessed racism experienced ever by 91.5% of students and 90.7% of physicians; discrimination ever experienced by 85.9% of students and 89.5% of physicians; bullying ever by 66.5% of students and 89.3% of physicians; harassment ever by 39.5% of students and 66.7% of physicians; majority witnessed Māori patients or whānau treated badly in clinical settings; 48% of students and 61.8% of physicians had considered leaving medicine; 25% of students and 37% of physicians had considered or taken a break specifically because of racism, discrimination, bullying or harassment; additional marginalised statuses were significantly associated with higher likelihood of direct mistreatment; women reported higher prevalence of most forms of mistreatment than men; junior physicians (graduated post-2011) reported higher exposure to racism than senior physicians	There is likely a selection bias as those with more experience of mistreatment or stronger Māori identity may have been more likely to respond, making results probable upper bounds; COVID-19 pandemic may have affected responses about the preceding 12 months; frequency of exposures not captured (only ever/last year); those on a break from or who had left medicine were not eligible to participate, likely underestimating true burden	New Zealand context provides closely comparable Indigenous-specific prevalence data on racism and mistreatment across the medical training and career continuum, directly informing understanding of similar issues likely experienced by Aboriginal and Torres Strait Islander medical trainees and physicians in Queensland
Curtis et al. (2025) New Zealand	Health professionals and healthcare organisations across all disciplines; Indigenous (Māori) and all culturally diverse patient populations	Definitions and conceptual frameworks for cultural safety and cultural competency; Indigenous health; health equity; medical regulation and accreditation	Refinement of 2019 cultural safety definition; review of MCNZ and Dental Council of New Zealand professional standards; proposed 2025 updated definitions of cultural safety and cultural competency	Revised, clearer definitions of cultural safety and cultural competency as related but distinct concepts; seven-point framework for practical cultural safety; four-point framework for practical cultural competency; seven key attributes for Indigenous health practice; seven synthesis lessons for international audiences	Cultural safety is frequently misunderstood or conflated with cultural competency in practice; cultural competency has been inappropriately dropped rather than maintained as a distinct domain; both concepts are needed and apply to all cultural groups, not only Indigenous peoples; cultural safety must explicitly foreground power and privilege (updated in 2025 definition); organisational responsibility for cultural safety remains under-implemented; assessment tools remain underdeveloped and over-reliant on self-reflection; cultural safety is politically vulnerable, particularly in a regressive NZ political climate; there is a need to move beyond individual practitioner responsibility to culturally safe organisations	Expert reflection rather than empirical study; primarily drawn from NZ context; definitions remain contested internationally; political resistance threatens implementation; assessment and measurement tools are underdeveloped	Provides the most current internationally recognised definitional framework for cultural safety and cultural competency, directly applicable to Queensland GP training, specialist vocational training, and Aboriginal and Torres Strait Islander health workforce development
Department of Health, Disability and Ageing [DHDA]	AGPT program participants: GP and Rural Generalist	Programs and initiatives; workforce	Australian General Practice Training (AGPT) Program 2026–2030,	Performance indicators and targets across four objectives: fellowship rates (≥80%); Aboriginal	Only 1% of doctors are Aboriginal and/or Torres Strait Islander against a 3.3% population share; projected national GP shortfall of 7,700 by 2033	Framework is prospective; targets are benchmarked against 2025 baseline data yet to be published; quantitative	National framework governs the AGPT program within which all Queensland GP

(2025) Australia	registrars, supervisors, training practices, Aboriginal and Torres Strait Islander registrars, and delivering organisations (ACRRM, RACGP, JCTS, IGPTN, CECMN, GPRA, GPSA)	representation and trends	involving the National Consistent Payments framework, Aboriginal and Torres Strait Islander Salary Support Program, and distribution policy	and Torres Strait Islander registrar enrolment (average 3% over rolling 3-year period); FTE training weeks in ACCHSs/AMSs (6% in 2026 rising to 8% by 2030); 100% of fellowing registrars and supervisors completing cultural education; at least 50% of training in MM 2–7 locations	rising to 12,400 by 2048; JCTS assigned shared accountability for cultural education delivery and cultural safety outcomes; escalating ACCHS/AMS placement targets signal increasing demand on Aboriginal and Torres Strait Islander training infrastructure; achieving Aboriginal and Torres Strait Islander enrolment targets requires more than doubling the 1.5% rate documented in 2025	targets do not address governance or self-determination requirements; no mechanism specified for Aboriginal and Torres Strait Islander community oversight of target achievement	registrars, and that includes Aboriginal and Torres Strait Islander registrars; ACCHS/AMS placement targets and Aboriginal and Torres Strait Islander enrolment targets directly applicable to Queensland workforce planning
Giddings et al. (2025) Australia	Non-specialist doctors (predominantly International Medical Graduates, 82% since 2013) already working in rural, remote, and First Nations communities; 506 enrolled RVTS participants across 350 communities; 10 of 32 annual places in Aboriginal Medical Services communities	Rural and remote GP workforce retention; distributed medical education; remote supervision; First Nations community health; International Medical Graduate training; workforce mal-distribution	Remote Vocational Training Scheme (RVTS): 25-year program providing distance education & remote supervision for non-specialist doctors to train toward RACGP/ACRRM fellowship while remaining in same rural/remote/First Nations community (~4 years)	Program completion and specialist certification rate; retention in same practice location during and after training; participant satisfaction; cost-effectiveness compared to similar rural training schemes; identification of five retention mechanisms (comfort, confidence, competence, belonging, bonding)	78% program completion and specialist certification rate; participants remained in the same practice for a mean of 5.2 years (approximately three times longer than typical retention for this rurality); 49% still working in the same community 2 years after completing the program (exceeding the high-performing benchmark of 30%); satisfaction levels 88–100% across various indicators; cost-effective compared to similar rural training programs; program supports First Nations community workforce continuity through 10 dedicated places annually in Aboriginal Medical Services; five participant responses identified as drivers of retention: comfort, confidence, competence, belonging, and bonding	Perspective article with inherent author conflict of interest (lead author employed by RVTS Ltd.); outcomes not directly comparable to other distributed rural training programs given RVTS's unique focus on retention in the same location rather than rotational placements; small annual intake (32 trainees) limits scale; digital divide in remote areas is a potential barrier; program primarily attracts IMGs, making domestic trainee retention outcomes harder to isolate	The University of Queensland led the 2023–24 independent evaluation; 15% of RVTS communities are medium-sized rural towns and many are in Queensland; the 10 annual First Nations community places are directly relevant to Queensland's Aboriginal and Torres Strait Islander health workforce and rural/remote GP retention challenges
Giddings et al. (2024) Australia	Non-specialist doctors (predominantly IMGs >80% since 2013) in rural/remote & First Nations communities; 506 total participants: Remote Stream (MMM4–7, n=434) & AMS Stream (MMM2–7, n=71); median age 36–40 yrs	Rural & remote GP workforce retention; distributed medical education; First Nations community health; AMS workforce; IMG workforce; Closing the Gap	RVTS (25yr, Australian Government-funded): remote supervision & distance education enabling RACGP/ACRRM fellowship while remaining in same practice 3–4 years; AMS Stream commenced 2013 with 10 dedicated annual places in	Cohort characteristics by stream, rurality, state & graduate type; AMS Stream participant distribution; alignment with 8 national rural workforce strategies	506 participants across 350 communities in all states/territories; Queensland: 2nd largest Remote Stream share (27.4%) & largest AMS Stream share (38%, n=27); 82.7% in MMM5–7; >80% IMGs since 2013; RVTS uniquely differentiated by retention focus & longer training period; informs 8 national policies including Closing the Gap	Perspective article. First Nations doctors not separately identified in the administrative dataset, limiting Aboriginal and Torres Strait Islander-specific analysis; AMS Stream places not always filled due to fewer applicants in some years; outcomes not comparable to other GP training programs given RVTS's unique retention design; small annual quota (approximately 32 trainees) limits overall scale	Queensland hosts the largest share of RVTS AMS Stream participants (38%, n=27 communities) and the second largest share of Remote Stream participants; the University of Queensland Rural Clinical School led the independent evaluation; program outcomes directly inform Queensland's Closing the Gap and rural/remote

			rural Aboriginal Medical Services				Aboriginal and Torres Strait Islander health workforce strategies
IGPTN (2025) Australia	Self-identified Aboriginal and/or Torres Strait Islander General Practice and Rural Generalist Trainees and Fellows engaged with IGPTN nationally	Workforce pipeline and support; cultural safety in GP/RG training; peer mentoring and education; membership growth; advocacy and representation; organisational governance	IGPTN program delivery: bi-annual face-to-face workshops, weekly online study and exam preparation groups, one-on-one mentoring, mock exams, peer yarning circles, and Fellowship awards	Membership numbers by state/territory and by college (RACGP vs ACRRM); membership growth over time (December 2023 – projected September 2025); workshop delivery (2 per year); member satisfaction (National GP Registrar Survey); organisational financial performance	Membership growing consistently from December 2023 to mid-2025, approaching and exceeding 100 total members; QLD has the highest membership by state/territory; RACGP members outnumber ACRRM members; two successful workshops delivered (Melbourne Oct 2024; Adelaide May 2025); total revenue $904,152; total expenses $678,809; surplus $225,343; net assets $357,753; high member satisfaction reported in the National GP Registrar Survey	Membership figures include Trainees and Fellows across all stages but do not capture non-members or the full national Aboriginal and Torres Strait Islander GP/RG workforce; financial data reflects inaugural year as an independent incorporated body (from July 2024); no disaggregation by gender or detailed career stage breakdown provided	Queensland has the largest membership by state/territory per the Members by State/Territory chart; findings directly relevant to Aboriginal and Torres Strait Islander GP workforce development and cultural safety in training across Queensland
Joint Colleges Training Services [JCTS] (2025) Australia	GP registrars and participants (RACGP and ACRRM members), GP supervisors, Aboriginal and Torres Strait Islander GP registrars, ACCHSs, Aboriginal and Torres Strait Islander communities	Programs and initiatives; barriers, enablers, and cultural safety frameworks	JCTS cultural education and mentorship program: Cultural Education Workshops, Cultural Observation Visits, Cultural Mentor Workshops, ACCHS Practice Manager Workshops, Roving Registrar Program, and Aboriginal and Torres Strait Islander GP registrar support; delivered under AGPT grant agreements on behalf of ACRRM and RACGP	Organisational growth from 26 to 41 staff over two years; service delivery across all states and territories; six strategic themes operationalised: cultural safety and responsiveness, cultural education excellence, community focus, wellbeing at work, strong partnerships, and resource efficiency	JCTS is the sole national body mandated to deliver cultural education across both GP colleges; strategic priorities include preparation and protection of Aboriginal and Torres Strait Islander registrars in mainstream placements, growing the Aboriginal and Torres Strait Islander cultural mentor workforce, and expanding ACCHS training capacity; governance comprises an independent Aboriginal and Torres Strait Islander Governing Board Chair alongside college executive representatives; core values include cultural integrity, reciprocity, and self-determination; TAFE NSW partnership established to deliver the Diploma of Practice Management for Aboriginal Medical Services	Inaugural strategic plan with no published outcome evaluation data; governance structure is a joint college venture rather than an Aboriginal and Torres Strait Islander community-controlled organisation; strategic plan is prospective without reported baseline performance metrics against each theme	JCTS operates regionally across Queensland; its mandate to deliver cultural education to 100% of AGPT registrars and supervisors directly affects the cultural safety environment for Aboriginal and Torres Strait Islander GP registrars training in Queensland
Koea et al. (2021) International (involving many countries but we focused on New Zealand as a comparable context)	Ethnic minority and Indigenous medical and surgical trainees and students globally; focus groups included African-Americans, Hispanic Americans, women identifying as ethnic minorities,	Affirmative action programmes (AAP); specialist postgraduate medical and surgical training selection; Indigenous and ethnic minority workforce	AAPs: equity-targeted admissions, ring-fenced positions, preparatory courses, mentorship, pipeline initiatives secondary school to leadership; examples include US Diverse Surgeons Initiative, RACS ring-fenced	Thematic synthesis yielding four themes: (1) need for social responsiveness in clinical training agencies; (2) justifications and criticisms of AAP; (3) how training agencies should participate in AAP; (4) what constitutes an effective	AAP effective at increasing ethnic minority undergraduate graduates but not highly assessed at specialist postgraduate level; under-representation persists with minimal progress in 20 years; Diverse Surgeons Initiative: 100% residency completion, 87% fellowship progression, 18% surgical leadership; 'pepper potting' risks cultural assimilation; all 7 Indigenous AAP studies affirmed their importance	All included studies were predominantly retrospective audits with heterogeneous outcomes, preventing quantitative synthesis; thematic analysis was applied to already-interpreted data rather than raw primary data, limiting derivation of pure themes; only seven studies addressed Indigenous-specific AAP experiences; evidence on AAP	International and New Zealand context; directly applicable to Queensland through the Royal Australasian College of Surgeons' Aboriginal and Torres Strait Islander surgical training initiative referenced in the paper; provides the only available evidence synthesis on AAP design

	New Zealand Māori, and people with low socioeconomic status; 45 articles included in final synthesis	representation; pipeline programs; mentorship; social accountability of training organisations	Indigenous surgical posts, NZ Māori admissions pathways	AAP for specialist medical and surgical training; a proposed pipeline-to-leadership AAP framework		efficacy in specialist postgraduate training specifically is absent; risk of publication bias in included literature	for specialist postgraduate training, including the proposed pipeline-to-leadership framework relevant to Queensland Indigenous GP and specialist workforce development
Martin & Reath (2011) Australia	GP registrars; Aboriginal and Torres Strait Islander communities; cultural educators and mentors; Regional Training Providers (RTPs); NACCHO affiliates	GP vocational training in Aboriginal and Torres Strait Islander health; curriculum development history; workforce partnership; cultural education and mentoring	GPET Framework for General Practice Training in Aboriginal and Torres Strait Islander Health; RACGP and ACRRM curricula; Aboriginal and Torres Strait Islander Health Training Posts; cultural educator and mentor roles within RTPs	Increased number of GP registrars undertaking Aboriginal and Torres Strait Islander health training placements; mandated training in both RACGP and ACRRM curricula; improved data collection; establishment of Aboriginal and Torres Strait Islander registrar liaison officer position	RACGP was the first Australian medical college to mandate Aboriginal and Torres Strait Islander health training in 1997; training placements grew substantially following the 2003 GPET Framework; Aboriginal and Torres Strait Islander health training posts (84% in ACCHSs by 2005) were financially supported and provided high-quality training environments; cultural educators and mentors are central to effective training but face barriers including lack of training, remuneration, and recognition; sustained partnership with Aboriginal and Torres Strait Islander people and organisations is the key determinant of success; increased training numbers risk quality dilution without further investment in capacity	Narrative review without systematic methodology; limited outcome data on clinical or health equity impacts; variation in RTP implementation not fully addressed; evidence base for specific training approaches was emerging at time of writing	Directly relevant to Queensland GP training history and current vocational training in Aboriginal and Torres Strait Islander health; highlights the importance of NACCHO-affiliate partnerships, ACCHS training posts, and CE/CM roles, all of which are applicable to Queensland's regional and remote contexts
Medical Board of Australia and AHPRA (2022) Australia	191 self-identified Aboriginal &/or Torres Strait Islander doctors in training nationally (from 22,135 eligible); interns 8%, prevocational 28%, specialist non-GP 33%, specialist GP 31%; 53% female; avg PGY 5.2; 96% Australian primary degree	Training quality; workplace culture; bullying, harassment, discrimination and racism; clinical supervision; assessment; teaching access; patient safety; career intentions; workforce wellbeing	Not applicable	Quantitative data on training experience across multiple domains, compared to national trainee averages	Aboriginal and Torres Strait Islander trainees consistently rated their training experience below the national average across all domains. Overall satisfaction was lower, with 64% recommending their training position compared to 78% nationally, and 70% recommending their workplace compared to 77% nationally. 55% of Aboriginal and Torres Strait Islander trainees experienced and/or witnessed bullying, harassment, discrimination, or racism, compared to 34% nationally; 70% did not report it, 29% considering leaving medicine (vs 20% national); 65% received cultural safety training (vs 77% national); 89% interested in Aboriginal & Torres Strait Islander health (vs 49% national)	Small Aboriginal and Torres Strait Islander subsample (n=191) limits subgroup analysis; some cells suppressed due to insufficient numbers; self-reported data subject to response bias; no qualitative data to explain observed disparities; national survey design not tailored to Aboriginal and Torres Strait Islander trainee experience; results not disaggregated by state/territory or specialty for this subgroup	Provides national benchmark data on the training experiences of Aboriginal and Torres Strait Islander doctors in training, with direct implications for Queensland medical colleges, hospitals and GP training organisations seeking to improve cultural safety, reduce racism, and support Aboriginal and Torres Strait Islander trainee retention and wellbeing
Medical Board of Australia and AHPRA	118 self-identified Aboriginal and/or Torres Strait	Medical training experiences;	Not applicable	Trainee-reported ratings across orientation quality, clinical	56% of Aboriginal and/or Torres Strait Islander trainees experienced and/or witnessed bullying, harassment,	Small sample size (n=118) limits subgroup analysis; some results suppressed due to bases	Survey is national but James Cook University (Queensland) was the

(2025) Australia	Islander doctors in training, drawn from 17,622 eligible respondents nationally; cohort includes interns (14%), prevocational/unaccredited trainees (24%), specialist non-GP trainees (26%), specialist GP trainees (31%), and international medical graduates (5%)	workplace culture and safety; bullying, harassment, discrimination and racism; clinical supervision; training curriculum; assessment; orientation; teaching access; patient safety; overall satisfaction; future career intentions; workforce retention		supervision quality, teaching quality, patient safety training quality, overall satisfaction (recommend training position/workplace), experience of bullying/harassment/discrimination/racism, workload, work-life balance, career intentions, and intention to leave medicine	discrimination, and/or racism (vs. 30% nationally); 38% reported experiencing and/or witnessing racism specifically; overall satisfaction lower than national average (73% would recommend training position vs. 83% nationally; 76% would recommend workplace vs. 83% nationally); 17% considering a career outside medicine within 12 months (vs. 9% nationally); 42% concerned about not successfully completing their training program (vs. 29% nationally); average working hours 47.4 hrs/week vs. national 44.0 hrs/week; 75% intend to work in Aboriginal and Torres Strait Islander health/healthcare; quality of orientation, teaching, supervision, and patient safety training all rated lower than national averages; 95% completed primary medical degree in Australia; postgraduate year average 4.8 years (vs. 5.7 nationally)	under n=10; data are unweighted; self-reported survey subject to response bias; 36.7% response rate nationally means non-response bias possible; single time-point cross-sectional design limits causal inference	medical school of origin for 12% of Aboriginal and/or Torres Strait Islander interns; 40% of the cohort trained in regional areas and 16% in rural areas, reflecting workforce distribution patterns relevant to Queensland; findings on racism, workload, and career attrition risk are directly applicable to Queensland health workforce planning and cultural safety policy
Milroy & Bandler (2023) Australia	GP supervisors and GP trainees (registrars); focus on Aboriginal and Torres Strait Islander health education in general practice settings	Decolonisation in medical education; antiracism in GP training; supervisor role modelling; cultural safety; Aboriginal and Torres Strait Islander health curriculum	Practical framework for GP supervisors applying decolonised and antiracist approaches; includes personal, collegial, supervisor, and curriculum-level strategies; two illustrative case studies of supervisor–trainee interactions	Conceptual framework and practical tools for GP supervisors; antiracism conversation prompts and starters; case studies for critical analysis of racism in clinical training settings	GP supervisors are pivotal in shaping trainees' cultural safety attitudes through mentorship & role modelling; racism is pervasive across all levels (health professional–patient, professional–professional, system levels); community placements in ACCHSs shift student perceptions; GP educators lack confidence delivering antiracist content	Commentary/discussion paper only. No primary data collected; recommendations are based on existing literature and authors' expertise rather than empirical study of supervisor behaviour or trainee outcomes; generalisability limited by lack of evaluation data	National Australian GP training context; highly applicable to Queensland GP supervisors and training organisations seeking practical guidance on embedding decolonised and antiracist approaches in supervision and curriculum
Milroy & Frayne (2025) Australia	Self-identified Australian Aboriginal and Torres Strait Islander general practice (GP) registrars undertaking GP training nationally, including metropolitan and	Racism experiences; cultural safety; professional wellbeing; training barriers and facilitators; Aboriginal and Torres Strait Islander health workforce	Not applicable	MIRE questionnaire responses across interpersonal, internalised, systemic, and race-consciousness domains; thematic analysis of interview data yielding three themes: (i) cultural identity and professional wellbeing/goals; (ii)	Aboriginal and Torres Strait Islander GP trainees experienced racism across multiple levels throughout training and workplace settings; cultural identity was central to professional goals and wellbeing; lack of cultural safety was pervasive; training facilitators included culturally safe placements (especially Aboriginal Community Controlled Health Organisations), dedicated support, mentorship, and Aboriginal and	Small sample size (n=11 survey, n=3 interviews) limiting generalisability; MIRE designed for patient cohorts and face-to-face delivery, reducing validity in this context; interviews could not be conducted face-to-face; potential confirmation bias from topic-specific recruitment; survey lacked specificity for	National Australian study; findings on racism, cultural safety barriers, and workforce facilitators are applicable to the Queensland Aboriginal and Torres Strait Islander GP training context

	rural trainees and those awaiting Fellowship; n=11 questionnaire, n=3 interviews			training challenges including racism and career development; (iii) training facilitators including specific support, placements, and mentorship	Torres Strait Islander peer networks; adaptive responses to racism included avoidance and peer support; all participants felt good about being Aboriginal and Torres Strait Islander despite challenges	healthcare workplace settings; those opting into interviews may have had higher racism exposure	
NACCHO (2024) Australia	Aboriginal and Torres Strait Islander communities; Aboriginal Community Controlled Health Organisations (145 members); GP registrars in rural and remote settings; international medical graduates	GP workforce distribution; rural and remote workforce pipeline; ACCHO workforce capacity; Medicare reform; GP registrar training pathways; international medical graduate policy; Closing the Gap Priority Reforms	Working Better for Medicare Review; National Agreement on Closing the Gap; Distribution Priority Area (DPA) framework; 10-year moratorium (19AB) for international medical graduates; GP registrar rural placement requirements	Seven policy recommendations covering: GP workforce incentives; pay parity and entitlement portability for registrars; rural/remote placement expansion; ACCHO supervisory capacity building; DPA boundary reform (MM4–MM7 only); 19AB moratorium exemption for all ACCHOs	94.4% of ACCHOs face recruitment challenges; 69.6% face retention challenges; NT GP registrar numbers fell from 61 (2016) to fewer than 10 (2023); only 13–19% of medical students prefer GP; 2022 DPA changes caused a 56% increase in doctors leaving regional Australia; ACCHOs provide 3.1 million episodes of care annually for over 410,000 people	Advocacy document rather than peer-reviewed research; workforce census data not publicly detailed; relies partly on third-party reports and anecdotal evidence from members; no quantitative outcome evaluation of proposed recommendations	National submission with specific reference to Far North Queensland as a region where ACCHO retention challenges are most acute due to inability to compete with government and private employer pay and conditions
Nychuk et al. (2024) Canada	First Nations, Inuit, and Métis medical applicants and learners across all stages of medical education (UGME, PGME, and continuing professional development)	Indigenous medical learner recruitment, admissions, and transitions; cultural safety criteria and assessment; workforce underrepresentation	Toolkit of wise-practice recommendations for Canadian medical schools (pre-admissions to lifelong learning); cultural safety admissions criteria; Community Obligation Clause	Recommendations across five life-course stages; environmental scan findings across 18 Canadian medical schools on Indigenous-specific admissions structures, requirements, and residency considerations	Indigenous physicians represent less than 1% of Canada's physician workforce (760 of 93,985 in 2016); nearly all medical schools have some Indigenous-specific admissions process, but significant gaps remain, particularly at PGME level; no medical school reported Indigenous-specific residency seat reservations; cultural safety assessment in admissions is inconsistent nationally	Environmental scan relied on publicly available front-facing data only; self-identification data likely inflated due to methodology; PGME-level data particularly sparse; recommendations not yet evaluated for implementation outcomes	Transferable frameworks for wholistic admissions review, cultural safety criteria, life-course pipeline approaches, and community obligation policies applicable to Aboriginal and Torres Strait Islander medical education in Queensland
RACGP (2024a) Australia	Aboriginal and Torres Strait Islander GP trainees, Fellows, medical students, junior medical officers, cultural educators, mentors, and medical educators; non-Aboriginal and	Cultural safety; Aboriginal and Torres Strait Islander health education and training; workforce development and pipeline; decolonisation of medical	Principles-based national framework with four elements: Cultural and Health Education and Training; Cultural Safety; Workforce Development; and GP Training Pipeline, underpinned by 12	Structured outcomes, strategies, indicators, and responsibilities across all four elements; targets include achieving population parity (≥3.8%) of Aboriginal and Torres Strait Islander trainees and Fellows; embedding Aboriginal and Torres Strait Islander	As of June 2024, Aboriginal and Torres Strait Islander people comprise only 1.3% of RACGP trainees and 0.39% of Fellows — well below the 3.8% population parity target; identified barriers include institutional racism, financial hardship, limited role models, placement inflexibility, and insufficient Aboriginal and Torres Strait Islander leadership within RACGP	Not an empirical study; implementation-dependent; risk of overburdening the small existing pool of Aboriginal and Torres Strait Islander leaders; resourcing and evaluation not yet secured	Framework applies across all RACGP regions including Queensland; directly relevant to Queensland's remote and regional Aboriginal and Torres Strait Islander communities; GP Training Pipeline and ACRRM partnership

	Torres Strait Islander trainees and GP workforce (cultural safety components)	education; anti-racism; self-determination in medical college governance; GP training equity	guiding principles including sovereignty, decolonisation, Indigenisation, strengths-based approaches, trauma-informed practice, and Aboriginal and Torres Strait Islander data sovereignty	epistemologies in curriculum; expanding ACCHO/AMS placements; and reducing racism in training			address rural generalist workforce needs specific to Queensland
Reath et al. (2018) Australia	Cultural Educators (CEs) and Cultural Mentors (CMs), Medical Educators, General Practice Supervisors, GP Registrars, Aboriginal and Torres Strait Islander Health Training Post delegates, and representatives of key stakeholder organisations (n=95 interviewees, 55 survey respondents)	Cultural education and mentoring roles; Aboriginal and Torres Strait Islander health workforce; GP registrar training; employment and support of Aboriginal and Torres Strait Islander educators	CE/CM model within Australian General Practice Training; Regional Training Organisation-based cultural education and mentoring programs	Thematic findings across three domains: understandings of CE/CM roles; employment and support challenges; delivery and evaluation of cultural education and mentoring	CE/CMs play a central and highly valued role in face-to-face registrar education about local Aboriginal and Torres Strait Islander culture, history, and health impacts; cultural education provided baseline learning while mentoring was longitudinal and relationship-based; key challenges included finding suitably skilled and community-authorised people, inadequate and inconsistent remuneration (CMs often unpaid), unclear role boundaries, risk of burnout, limited professional development pathways, and insufficient evaluation; organisational support, respectful partnerships with Medical Educators, and community engagement were identified as essential for sustainability	Survey respondents unlikely to be a representative sample; CE/CM and Registrar survey responses could not be linked; findings reflect a single national training system at one point in time; CMs' contribution remains difficult to quantify and formally evaluate	Directly applicable to Queensland GP training contexts; identifies sustainable models for engaging Aboriginal and Torres Strait Islander CEs and CMs, with specific relevance to Queensland's regional, rural, and remote training posts serving Aboriginal and Torres Strait Islander communities
Reid et al. (2024) Australia	Aboriginal and/or Torres Strait Islander non-GP specialist trainees and prospective trainees engaged with AIDA's STSP; also includes AIDA staff and governance representatives, Commonwealth DoHAC representatives, and representatives from 13 non-GP	Indigenous non-GP specialist workforce pipeline; cultural safety in specialty training; racism & lateral violence; mentoring; financial barriers; program governance & self-determination	AIDA Specialist Trainee Support Program (STSP), initiated April 2022 to provide monthly trainee catch-ups, one-on-one performance coaching and advocacy, virtual Yarn Up sessions, trainee workshops, specialist trainee network establishment, and cross-college governance	Trainee and Fellow numbers by college over time; recruitment and retention rates; trainee and supervisor feedback on workshop quality and cultural safety; cross-college actions and benchmarks from IMST reports; attendance and engagement data from cultural safety webinars; qualitative experience data from trainees and stakeholders	Aboriginal and Torres Strait Islander Fellows & trainees in specialty colleges grew from 108 (Feb 2020) to 262 (Mar 2023) (142% overall; 240% in non-GP specialties); all colleges recently reported 100% retention; 100% STSP interview coaching success; 39% of Aboriginal & Torres Strait Islander trainees experienced bullying, harassment, discrimination, or racism; 52% witnessed incidents; STSP may be the only program of its kind internationally	Three-year data window limits long-term trend analysis; retention data may undercount trainees on leave due to racism or cultural distress; data collection inconsistencies across colleges; college funding siloes limit uniform STSP implementation; program scope does not currently extend into hospitals and clinical settings where most negative experiences occur; small STSP staff team creates vulnerability to disruption from turnover	The STSP operates nationally and includes Queensland-based trainees; findings on barriers (racism, financial hardship, inflexible training, isolation in specialty pathways) are directly applicable to Queensland's Aboriginal and Torres Strait Islander specialist medical workforce context; Goondir Health Service (St George, QLD)

	specialist medical colleges		coordination across 13 non-GP specialist colleges				referenced as a trainee placement site
Rourke et al. (2018) Canada	MD students and postgraduate Family Medicine residents at Memorial University graduating classes 2011–2020; subset of self-identified Aboriginal students (n=23)	Rural medical workforce pipeline; Aboriginal and Torres Strait Islander recruitment and admissions; rural-focused medical education; postgraduate rural training; continuing professional development; social accountability	Multi-stage 'pathways to rural practice' model: MedQuest school outreach; Aboriginal Initiative (school visits, Healers of Tomorrow Gathering, Pre-Med Summer Institute, MCAT Grant, 3 reserved Aboriginal MD seats); holistic rural-weighted admissions; rural pre-clerkship/clerkship placements; 8-week mandatory Rural FM clerkship; Family Medicine residency rural stream; NORFAM stream (21 months in Labrador)	Geographic background of students; proportion of placement weeks in rural locations across MD and PGME training; rural practice rates post-graduation; Aboriginal student enrolment and geographic background	56% of MD-graduating classes 2011–2020 had rural backgrounds; 23 self-identified Aboriginal MD students as of September 2016, and of the 22 with recorded background locations, 91% were from rural locations; 90% of Year 1 Community Health placements were in rural settings; 89% of Year 3 Rural Family Medicine placement weeks in rural communities; 100% of Family Medicine residents (2011–2013 cohort, n=49) completed some rural training, averaging 52 weeks rural out of 95 weeks total; 26.9% of Memorial Family Medicine graduates practicing rurally 2 years post-training vs national average of 13.3%	Administrative data only; no comparator institution in this paper (companion paper provides comparative analysis); geospatial classification system is MUN-specific and may not be directly transferable; Aboriginal student numbers are small; data currency limited to 2016 for Aboriginal enrolment	Canadian context offers a well-evidenced, transferable model for end-to-end rural generalist pipelines including Aboriginal and Torres Strait Islander-specific pre-admission outreach, reserved seats, holistic admissions, longitudinal rural placements, and rural residency streams applicable to Aboriginal and Torres Strait Islander medical education and rural workforce planning in Queensland
Taylor et al. (2024) Australia	1,373 GP registrars in active training during Semester One 2024 across AGPT (91%), RGTS (7%), and RVTS (2%) programs; 35.5% response rate from a target population of 3,870	GP training satisfaction; workforce demographics; training context; income and remuneration; rural generalist pathway; Aboriginal and Torres Strait Islander health training; career intentions; health and wellbeing	Not applicable	Registrar satisfaction with training providers (GP Colleges/RVTS) and training facilities; Key Performance Indicator (KPI) metrics; income and support payments; rural generalist pathway engagement; cultural awareness training participation; professional membership engagement; career plans	89% satisfied with overall training from training provider; 91% satisfied with training facility overall; 96% satisfied with clinical work, patient numbers, diversity, and workplace responsibility; 55% earn less in GP training than in prior prevocational hospital role; 53% of full-time registrars reported median income $50,000–$59,999/semester; 88% had participated in Aboriginal and Torres Strait Islander cultural awareness training (up 13 percentage points from 2023); 70% knew how to access a cultural mentor; 2.3% identified as Aboriginal or Torres Strait Islander; 79% of Rural Generalists intended to remain in rural practice	Self-report survey with 35.5% response rate; non-mandatory questions reduce completeness for some items; RVTS registrar data is deidentified limiting follow-up; does not capture registrars on extended leave; income data cannot be verified independently; small subgroup numbers (e.g. RVTS, IGPTN) limit subgroup analysis	National dataset with some state-level income breakdowns; Queensland registrars (32%) more likely to earn more than in prevocational training than Victorian counterparts (12%); Queensland Rural Generalist Pathway Coordination Unit engaged by 91% of Queensland RG registrars; no Queensland-specific satisfaction or demographic breakdown provided separately
Taylor et al. (2025)	1,225 GP registrars enrolled in active	Registrar satisfaction with	Australian General Practice Training	Satisfaction scores (5-point scale) with training	92% satisfied with overall training from training provider (historic high); 92%	Self-selected survey respondents (31.1% response	national data with no state-level breakdown

Australia	Commonwealth-funded GP training (AGPT, RGTS, RVTS); 31.1% response rate from a target population of 3,939	training providers and facilities; workforce demographics; rural and remote training; Aboriginal and Torres Strait Islander health training; Rural Generalist pathway; Single Employer Model trials; multidisciplinary team integration; career intentions	(AGPT); Rural Generalist Training Scheme (RGTS); Remote Vocational Training Scheme (RVTS); Single Employer Model (SEM) trials	providers and facilities; Key Performance Indicator (KPI) rates; cultural training participation; career plan distributions; SEM trial experience; multidisciplinary team working; relocation rates	satisfied with training facility; 97% satisfied with clinical work; 25% of respondents are Rural Generalist registrars; 43% are International Medical Graduates; 65% female; 2.3% identify as Aboriginal or Torres Strait Islander; 87% completed cultural awareness training; Rural Generalists >4× more likely to plan rural/remote work and >3× more likely to plan hospital-based work than non-RG peers; SEM trial registrars reported 96% agreement it provided training diversity and 86% said it met or exceeded expectations; 70% of non-SEM registrars were unaware the SEM arrangement existed	rate, down from 35.5% in 2024); data unweighted; small cell sizes suppressed for confidentiality; Aboriginal and Torres Strait Islander subgroup (n=28) too small for reliable disaggregated analysis; RVTS response rate lower than national average (23.2%); reflects Semester One 2025 only	reported in the main findings; Queensland SEM registrars represented (n=20, 23% of SEM respondents); Queensland Rural Generalist Pathway Coordination Unit was the most engaged state unit (28% of RG registrars)
Tipene-Leach et al. (2024) New Zealand	Medical practitioners (registrars and specialist vocational trainees); Māori patients and communities	Cultural safety training frameworks; cultural competence; Māori health equity; medical education policy	Cultural Safety Training Plan for Vocational Medicine in Aotearoa; MCNZ cultural safety standards; CMC vocational training framework	Development and description of a cultural safety training framework with four core proficiencies, five enabling sub-proficiencies each, suggested teaching methods, and assessment/CPD tasks	Cultural safety training for NZ medical practitioners evolved from cultural sensitivity through cultural competence to cultural safety over three decades; the 2019 MCNZ Statement on Cultural Safety replaced the 2006 competence statement; research found cultural safety training was underdeveloped in vocational colleges, Māori trainees felt culturally unsafe, and safety was supervisor-dependent; a structured training plan was developed centred on critical consciousness, power relationships, transformative action, and patient-defined safety; significant challenges remain including an internationally trained workforce, limited evidence base, and cultural load on Māori practitioners	Limited evidence base linking cultural safety training to improved health outcomes; large proportion of workforce is internationally trained limiting reach of vocational CPD; evaluation of the training plan is still needed; cultural load on Māori educators and practitioners not yet adequately addressed	NZ cultural safety framework offers a transferable model for Aboriginal and Torres Strait Islander health equity training in Queensland general practice and specialist vocational training, particularly the proficiency-based structure and self-reflective practice emphasis
Worley et al. (2019) Australia	Medical students undertaking the Northern Territory Medical Program (NTMP) from 2011 to 2016; n=144 enrolled students including 20 Aboriginal and	Aboriginal and Torres Strait Islander medical workforce development; distributed medical education; rural	NTMP: full 4-year distributed medical degree (Darwin, 2011–), contextualised to remote/tropical/Aboriginal and Torres Strait Islander health; 3 entry	Graduation rates; NT retention post-graduation; Aboriginal and Torres Strait Islander student enrolment and graduation proportions; examination performance compared with Adelaide cohort;	First cohort graduated December 2014 (8 of 24 starters); 22 graduated in 2015 and 19 in 2016; 75% of the first cohort remained in the NT after completing their return-of-service obligation; examination scores post-2011 statistically indistinguishable between Darwin and Adelaide cohorts; 14% of entrants and 14% of graduates were	Short time frame of reporting (2011–2016) limiting long-term outcome assessment; first cohort was atypical of subsequent cohorts with lower science literacy and older mean age; Aboriginal and Torres Strait Islander student progression and graduation	Northern Territory context is highly relevant to Queensland given comparable remote, rural, and Aboriginal and Torres Strait Islander health workforce challenges; demonstrates feasibility and outcomes

	Torres Strait Islander students (14 NT residents); NT residents and Aboriginal and Torres Strait Islander Australians given priority in selection	and remote workforce recruitment and retention; social accountability of medical schools; Aboriginal and Torres Strait Islander student pipeline	pathways: graduate entry; secondary school entry (BCS/BMBS double degree with Charles Darwin University); Aboriginal and Torres Strait Islander Entry Stream with dedicated preparatory program; all places fee-free & bonded to NT Government for 4 years post-graduation	program development lessons	Aboriginal and Torres Strait Islander which is the highest proportion of any Australian medical school at the time; 20 Aboriginal and Torres Strait Islander students recruited and 7 graduated between 2011 and 2016; 11 Aboriginal and Torres Strait Islander staff employed in support roles; Aboriginal and Torres Strait Islander Entry Stream preparatory program substantially revised over 4 years to improve rigour and progression outcomes	rates were initially challenging; single-institution program report without comparison group; no formal evaluation framework applied	of prioritised Aboriginal and Torres Strait Islander entry streams and distributed medical education in an analogous Australian jurisdiction
