# Supplementary material for: Strengthening the pipeline of Aboriginal and Torres Strait Islander doctors into and through general practice training in Queensland, Australia: a scoping review
Source: Front Med (Lausanne). 2026 Aug 24;13:1921854. doi: 10.3389/fmed.2026.1921854 (PMC13584867; doi:10.3389/fmed.2026.1921854)
Supplement: Supplementary file 2 [file Data_Sheet_2.pdf]

Supplementary Table. Search strings used across all databases

No.	Database/Source	Search string	Filters applied	Date(s) searched	Results (n)
1	**Scopus (Elsevier)**	(("Aboriginal Australian" OR "Torres Strait Islander"<br>OR "Indigenous Australian doctor"<br>OR "First Nations physician" OR "Indigenous physician"<br>OR "Maori doctor" OR "Indigenous medical trainee"<br>OR "Aboriginal doctor" OR "Indigenous doctor"<br>OR "Aboriginal medical graduate")<br>AND<br>("general practice training" OR "GP training"<br>OR "general practitioner training"<br>OR "vocational training" OR "primary care training")<br>AND<br>("workforce" OR "cultural safety" OR "racism"<br>OR "barriers" OR "enablers" OR "retention"<br>OR "recruitment" OR "mentorship" OR "leadership"<br>OR "pipeline"))	Date: 2016–2026; Language: English; Document type: Article, Review; Subject area: Medicine, Health Professions, Multidisciplinary, Social Sciences	11 March 2026	**79**
2	**PubMed/MEDLINE**	(("Aboriginal Australian" OR "Torres Strait Islander"<br>OR "Indigenous Australian" OR "Maori doctor"<br>OR "Aboriginal medical"<br>OR "Australian Aboriginal and Torres Strait Islander Peoples"[MeSH Terms]<br>OR "Health Services, Indigenous"[MeSH Terms])<br>AND<br>("general practice training" OR "GP training"<br>OR "general practitioner training"<br>OR "vocational training" OR "prevocational training"<br>OR "GP registrar"<br>OR "General Practice"[MeSH Terms]<br>OR "Education, Medical, Graduate"[MeSH Terms])<br>AND<br>("workforce" OR "cultural safety" OR "racism"<br>OR "barriers" OR "retention" OR "recruitment"<br>OR "mentorship" OR "leadership" OR "pipeline"<br>OR "Cultural Competency"[MeSH Terms]<br>OR "Health Workforce"[MeSH Terms]<br>OR "Racism"[MeSH Terms]))	Date: 2016–2026; Language: English; Humans; MEDLINE	11 March 2026	**105**
3	**CINAHL (EBSCOhost)**	(("Aboriginal" OR "Torres Strait Islander" OR "Indigenous Australian"<br>OR "Aboriginal and Torres Strait Islander" OR "Māori" OR "First Nations"<br>OR "Indigenous")<br>AND<br>("general practice training" OR "GP training" OR "general practitioner training"<br>OR "vocational training" OR "prevocational training" OR "GP registrar" OR "general practice registrar" OR "medical training" OR "medical education")<br>AND	Date: 2016–2026; Language: English	11 March 2026	**62**

		("workforce" OR "cultural safety" OR "racism" OR "barriers" OR "enablers" OR "retention" OR "recruitment" OR "mentorship" OR "leadership" OR "pipeline" OR "cultural competence" OR "cultural competency"))			
4	**Google Scholar**	"Aboriginal" OR "Torres Strait Islander" AND "general practice training" AND "cultural safety" OR "workforce" OR "racism" OR "barriers" OR "retention"	Date: 2016–2026; Peer reviewed	11 March 2026	**354**
5	**ProQuest**	("Aboriginal Australian" OR "Torres Strait Islander" OR "Indigenous Australian" OR "Aboriginal and Torres Strait Islander" OR "Maori" OR "First Nations" OR "Indigenous Canadian") AND ("general practice training" OR "GP training" OR "general practitioner training" OR "vocational training" OR "GP registrar" OR "general practice registrar" OR "primary care training") AND ("workforce" OR "cultural safety" OR "racism" OR "barriers" OR "retention" OR "recruitment" OR "mentorship" OR "leadership" OR "pipeline") AND ("Australia" OR "New Zealand" OR "Canada")	Date: 2016–2026; Language: English; Document type: Article, Review, Literature Review, Evidence Based Healthcare; Peer reviewed	12 March 2026	**419**
6	**Informit Indigenous Collection**	("Aboriginal" OR "Torres Strait Islander" OR "Indigenous Australian" OR "Aboriginal and Torres Strait Islander" OR "First Nations") AND ("general practice" OR "GP training" OR "general practitioner" OR "primary care" OR "primary health care" OR "medical training" OR "medical education" OR "vocational training") AND ("workforce" OR "cultural safety" OR "racism" OR "barriers" OR "retention" OR "recruitment" OR "mentorship" OR "leadership" OR "pipeline" OR "cultural competence")	Date: 2016–2026; Language: English; Peer reviewed where available	12 March 2026	**85**
7	**Grey literature (organisational & policy sources)**	Targeted searching of organisational websites and publication repositories (RACGP, ACRRM, AIDA, IGPTN, JCTS, NACCHO, Medical Board of Australia/ AHPRA and the Medical Training Survey, Australian Department of Health, Disability and Ageing, and equivalent Canadian and New Zealand bodies), plus targeted Google and Google Scholar searching and reference-list (citation) searching of included sources.	Date: 2016–2026; English; organisational and policy documents; searched separately from bibliographic databases	4 March to 30 April 2026	**66**

Note: MeSH terms were applied in PubMed/MEDLINE only. All searches were conducted in English. Date restriction applied: January 2016 – March 2026. Record counts are those retrieved on the dates of execution stated. Database indexing is dynamic, and re-execution at a later date may return a different number of records. Grey-literature sources were searched across multiple days, and a date range is therefore reported.
